# The effects of ageing on fatigue and endurance of the spinal extensor muscles: a systematic review and meta-analysis

**DOI:** 10.1007/s11357-025-01987-x

**Published:** 2025-11-08

**Authors:** Martina Parrella, Michail Arvanitidis, Andrea Macaluso, Deborah Falla

**Affiliations:** 1 Department of Movement, Human and Health Sciences, Università Degli Studi Di Roma “Foro Italico”, Rome, Italy; 2 Centre of Precision Rehabilitation for Spinal Pain (CPR Spine), School of Sport, Exercise and Rehabilitation Sciences, College of Life and Environmental Sciences, University of Birmingham, Birmingham, UK

**Keywords:** Ageing, Muscle fatigue, Endurance, Spinal extensor muscles, Back extensor muscles

## Abstract

The endurance capacity of the spinal extensor muscles plays a key role in maintaining spinal function. This systematic review and meta-analysis aims to synthesise current evidence on how ageing influences fatigue of the spinal extensor muscles, addressing the inconsistent findings reported across existing studies. Medline, EMBASE, PubMed, Web of Science and CINAHL Plus databases were searched from their inception to 28 June 2025. Cross-sectional studies assessing fatigue of the spinal extensor muscles of healthy older adults (>60 years) versus younger adults were included. Methodological quality was evaluated using the Appraisal Tool for Cross-Sectional Studies and the GRADE approach was applied to assess the certainty of evidence. Results were synthesised using both narrative and quantitative approaches. A random-effects meta-analysis was conducted for endurance time. The PRISMA guidelines were followed for reporting. Of the 1253 records screened, 13 studies were included, with 9 contributing to the meta-analysis. The meta-analysis, supported by moderate certainty of evidence, revealed a significant reduction in endurance time of the back extensor muscles during sustained isometric contractions in older adults compared to younger controls (MD = −41.31; 95% CI: −64.04; −18.57). The remaining outcomes were synthesised narratively: electromyograph (EMG) related measures showed mixed findings, likely due to methodological variability across studies, while force decline and Borg ratings were reported in only a few studies. This systematic review revealed that the endurance time of the back extensor muscles is reduced during isometric tasks in older adults. However, inconsistent EMG findings limit our understanding of the neuromuscular mechanisms underlying this decline.

**Supplementary Information:**

The online version contains supplementary material available at https://doi.org/10.1007/s11357-025-01987-x.

## Introduction

Optimal functioning of the spinal extensor muscles, which include the lumbar, thoracic and cervical (neck) extensors, is essential for spinal posture and function. As these muscles are usually exposed to sustained or repetitive low-level contractions to maintain upright posture, previous studies have emphasised the importance of their endurance capacity [1, 2]. Thus, muscle fatigue, defined as a reduction in the force/power-generating capacity of skeletal muscles in response to prolonged or repeated activity [3], has been widely investigated in both back and neck muscles, across healthy and clinical populations. For instance, it has been demonstrated that fatigue of the back extensor muscles can impair postural control during both static [4, 5] and dynamic tasks [6]. Additionally, reduced back muscle endurance has been identified as a predictor of long-term back-related disability [7]. Similarly, fatigue of neck extensor muscles has been associated with altered cervical position sense [8], and decreased endurance time has been previously reported in individuals with chronic neck pain [9].

Given the critical role of spinal extensor muscles for spinal health, their function has also been investigated in the context of ageing. Previous studies have demonstrated that both paravertebral and cervical extensor muscles undergo age-related degenerative changes, including muscle atrophy and increased fat infiltration [10, 11]. Interestingly, Beauchamp et al. [12] reported that trunk extensor endurance is among the key physical factors predicting participation in life roles in older adults. Improvements in trunk extension endurance have also been associated with enhanced balance in community-dwelling older adults with mobility limitations [13]. Moreover, as the prevalence of neck pain increases with age and has a substantial impact on quality of life [14], improving neck extensor endurance may be beneficial for older populations to prevent and/or reduce pain [15, 16].

Several studies have investigated how the endurance of spinal extensor muscles changes with ageing. Some authors have observed lower endurance time in older people when performing the Ito test [17] and the Biering-Sørensen back endurance test [18]. However, other studies have reported either increased endurance time during dynamic back extensions in the elderly [19] or no significant differences in trunk extension endurance between older and younger individuals during a modified Sørensen test [20, 21]. Additionally, some previous studies have employed surface electromyography (sEMG) to assess the myoelectric manifestations of fatigue in spinal muscles, typically by analysing the decline in median frequency (MF) and/or mean power frequency (MPF) over time. While some authors have reported a smaller decline in MF in older participants during sustained isometric back extensions [22], others found no significant differences during a back extension exercise using the Roman Chair [23], or reported a reduced decline only in older men, but not in women [24].

In light of inconsistent findings across the literature, this systematic review and meta-analysis aims to synthetise and critically evaluate existing evidence on whether the development of fatigue in the spinal extensor muscles and endurance time differs in older versus younger adults.

## Methods

This review is reported in line with the 2020 guidelines of Preferred Reporting Items for Systematic reviews and Meta-Analyses (PRISMA) (Supplemental file 1) [25]. The protocol for this review was registered on the International Prospective Register of Systematic Reviews (PROSPERO; CRD42025645295) on 31 January 2025.

### Eligibility criteria

The PICOS framework, which includes Population, Intervention, Comparison, Outcomes and Study design, was used to define the inclusion criteria for this systematic review. However, the original term “Intervention” was replaced with “Indicator” due to the nature of potentially eligible studies, as reported by previous similar systematic reviews [26, 27].

#### Population

Studies were considered eligible for inclusion if they involved physically independent older adults aged 60 years and above. Although the age cutoff for defining an “older” person may vary across countries, the 60+ threshold was selected based on the United Nations’ definition [28]. Additionally, studies were required to include a control group of young and/or middle-aged individuals as comparators. Only studies involving healthy participants were considered, defined as individuals free from musculoskeletal disorders, neuro-systemic degenerative diseases, chronic cardiovascular or respiratory system diseases. Participants also had to be free from a history of chronic spinal pain, current spinal pain, spinal surgery, or spinal deformities. Lastly, there were no restrictions in terms of gender and/or ethnicity.

#### Indicator

Eligible studies were those assessing muscle fatigue in the spinal extensor muscles and/or endurance, including lumbar, thoracic, and neck extensor muscles. Studies involving voluntary fatiguing contractions performed either for a fixed duration or until task failure were included. The tasks involved the use of a specific dynamometer for trunk muscles or a standardised test for spinal extensor endurance, such as the Biering–Sørensen test or the Ito test. The Biering–Sørensen test and its variations (i.e., modified Sørensen test on a Roman Chair) and the Ito test are standardised isometric tests designed to assess the endurance of the back extensor muscles [29]. In these tests, participants are positioned prone and instructed to lift the upper body to maintain a horizontal posture for as long as possible or for a predetermined duration. These tests differ primarily in the type of support (e.g., Roman chair or bench), fixation methods (e.g., lower body stabilisation) and arm positioning. All types of contractions were included (i.e., isometric or dynamic), measured both at an absolute force/torque level or relative to the individual’s maximal voluntary contraction (MVC).

#### Comparison

Eligible studies included comparisons of specific outcome variables (e.g., endurance time, EMG parameters) between groups, such as older versus younger participants or older versus middle-aged participants.

#### Outcomes

Outcome variables included any measure related to muscle fatigue development and endurance. For this purpose, both mechanical variables, such as endurance time and decline in force values between pre- and post-fatigue assessments, and neuromuscular variables, such as MF or MPF of the EMG signals and muscle fibre conduction velocity (MFCV), were considered.

#### Study design

Only cross-sectional studies were included in this systematic review, as this design is most commonly used to address the research question. Therefore, non-original literature, such as systematic and narrative reviews as well as other research designs such as randomised controlled trials, were excluded. To minimise the risk of bias, studies in all languages were included in the search. However, non-English studies were ultimately excluded due to time and resource constraints.

### Information sources

Electronic searches were performed on Medline (Ovid Interface), EMBASE (Ovid Interface), PubMed, Web of Science (Clarivate Analytics) and CINAHL Plus (EBSCO Interface) databases from their inception to 28 June 2025. Hand searching of key journals—including Journal of Physiology, Journal of Neurophysiology, Journal of Electromyography and Kinesiology, European Journal of Applied Physiology, Journal of Applied Physiology, Muscle & Nerve—was also conducted. Additionally, the reference lists of all included papers were checked manually to identify any additional relevant studies that could have been missed during the search.

### Search strategy

The search was conducted by the lead author (MP) without any restrictions in terms of date, geographical area and language. Search strategies were tailored for each database, and Medical Subject Headings (MeSH) were used when appropriate to optimise the search process. This approach combined MeSH terms and free-text keywords. Although the search was adapted for different databases (e.g., syntax), consistency was ensured. The full electronic search strategies for all databases are reported in Supplemental file 2.

### Selection process

All search results were imported in EndNote 20 (Clarivate Analytics) by the lead author (MP). Duplicates were identified and automatically removed by the software. All remaining references were then imported into Covidence (Veritas Health Innovation, Melbourne, Australia), where the title/abstract and full-text screening processes were conducted. Titles and abstracts of the studies were independently screened by two reviewers (MP and MA) using a pretested screening form. Studies were categorised as eligible, ineligible or doubtful. Doubtful studies were discussed between the two reviewers, and a third reviewer (DF) was involved in resolving any disagreements or uncertainties. After the initial screening phase, eligible studies underwent full-text assessment, which was always conducted independently by the same two reviewers (MP and MA), with the third reviewer (DF) assisting in case of disagreements. If the full-text of an eligible study could not be retrieved by the reviewers, the authors were contacted with a two-week reply window.

### Data collection process and data items

Data extraction was conducted by one reviewer (MP) using a pretested extraction form that was specifically designed to align with the review’s aims. A second reviewer (MA) verified the accuracy of the extracted data. If clarifications about the data were necessary (e.g., incomplete data), the authors were contacted with a two-week reply window. When data were available only in graphs or tables, WebPlotDigitizer (version 5.1) software was used to extract them [30]. Relevant data for each aspect of the PICOS framework were extracted. General study information (e.g., participant characteristics) and subjective measures of muscle fatigue (e.g., Borg scale ratings) were also reported. If studies included groups not relevant to the review’s aims, they were not considered for data extraction.

### Risk of bias assessment

Two independent reviewers (MP and MA) assessed the methodological quality of the included papers using the Appraisal tool for Cross-Sectional Studies (AXIS) [31]. The original version of the tool consists of 20 questions divided into five different sections: introduction, methods, results, discussion, and other. However, the tool was slightly modified to better align with the objectives of this review, as previously reported [32]. Specifically, questions 7 and 14 were removed, as non-response bias had little relevance in our review due to the nature of the included studies. Question 13 of the original tool (now renumbered as question 12 in the modified version) was revised to assess whether information about participant dropouts during testing sessions were reported. Each question was answered with “Yes”, “No”, or “Don’t know”. A response of “Yes” was awarded 1 point, while “No” or “Don’t Know” responses were awarded 0 points. However, question 19 of the original tool (now renumbered as question 17 in the modified version), which is related to the conflicts of interest, was scored differently. Due to the nature of the question, a response of “Yes” was awarded 0 points, while “No” received 1 point. The maximum possible score for each study was 18, with higher scores indicating lower risk of bias. To ensure comparability across studies, scores were converted into percentages. Studies scoring ≥ 75% were classified as “good” quality, those scoring between 50 and 74% as “moderate” quality, and those scoring <50% as “poor” quality [33].

### Synthesis methods

Both narrative and meta-analytic approaches were employed to synthesise the data from the included studies [34, 35]. The narrative synthesis was conducted for endurance time, EMG parameters, and force decline while the meta-analysis was performed for endurance time only. No grouping was necessary for the meta-analysis, as all studies included the same outcome measure (endurance time in seconds), allowing for direct comparisons. The narrative synthesis involved outlining and tabulating study characteristics to facilitate a comprehensive interpretation of findings across all studies [34]. For the meta-analysis, only studies with available or retrievable mean ± SD data on endurance times (in seconds) and sample sizes for both older and younger groups were included. The endurance results reported by Tsuboi et al. [24] were pooled into a single group, as the authors originally reported data separately for male and female participants. The pooled mean and standard deviation were calculated using the following formulas:

$$\mathrm{Mean}\_\mathrm{pooled}=\frac{\left(N \mathrm{male}\times \mathrm{mean}\_\mathrm{male}\right)+ \left(N \text{female }\times \mathrm{mean}\_\mathrm{female}\right) }{N \mathrm{male}+N \mathrm{female}}$$

$$\mathrm{SD}\_\mathrm{pooled}= \sqrt{\frac{\left[\left(N \mathrm{male}-1\right)\times {SD}^{2}\_\mathrm{male}\right]+\left[\left(N \mathrm{female}-1\right)\times {SD}^{2}\_\mathrm{female}\right] }{N \mathrm{male}+N \mathrm{female}-2}}$$

The mean difference (MD) was selected as the outcome measure, as endurance time in seconds was a common metric across studies.

Meta-analysis procedures and forest plot generation were performed using R software (version 4.4.3) [36] with the “meta” package (v. 8.0.2) [37]. A random-effects model was chosen to account for the anticipated heterogeneity across studies, common in ageing research due to variations in study protocols, participant characteristics (including age ranges and fitness levels), sample sizes, task specificity, and measurement techniques. The Knapp-Hartung adjustment was applied to calculate the 95% confidence intervals around the pooled effect estimate [38], providing more conservative estimates particularly suited for meta-analyses with smaller numbers of studies. Additionally, prediction intervals were included alongside summary estimates to illustrate the expected range of true effects in future studies. This approach enhances clinical interpretation by showing the breadth of potential outcomes rather than just the average effect [35, 39].

While a random-effects model was implemented to account for heterogeneity, it is important to acknowledge that this approach does not eliminate heterogeneity but accommodates it statistically [35]. Additional measures were taken to explore heterogeneity beyond the commonly used I^2^ statistic, which has limitations in fully characterising heterogeneity patterns. To gain further insight into the variation in true effects across studies, the between-study variance (τ^2^) was estimated using the restricted maximum-likelihood (REML) estimator, as recommended for continuous outcomes [35, 39]. Prediction intervals were also calculated for pooled effect sizes to offer a more comprehensive perspective on the potential range of true differences in endurance times between older and younger adults across studies [35, 39]. This approach enhances clinical interpretation by illustrating not only the average effect but also the expected range of effects in future studies.

### Sensitivity analyses

Given the anticipated heterogeneity in ageing studies comparing endurance measures, comprehensive sensitivity analyses were conducted [35]. This involved sequentially excluding each study to assess the consistency of the meta-analysis results and evaluate the impact of individual studies on overall heterogeneity. Outliers and influential cases were also quantitatively explored using established deletion diagnostics adapted from linear regression [40, 41]. These diagnostics included externally standardised residuals, DFFITS values, Cook’s distances, covariance ratios, DFBETAS values, estimates of τ^2^ and Q when each study was removed sequentially, diagonal elements of the hat matrix, and the weights assigned to observed outcomes during model fitting. These analyses were performed using the *“influence”* function from the *“metafor”* package (v. 4.6.0) in R [40]. The influence function allows for a quantitative identification of potential outliers and influential studies that may disproportionately affect the meta-analysis results, facilitating the detection of studies that could bias the findings. This comprehensive approach to identifying influential cases and conducting sensitivity analyses ensured the robustness of the findings regarding age-related differences in endurance times.

### Certainty of evidence

The overall certainty of the evidence was independently assessed by two reviewers (MP and MA) using the Grading of Recommendations, Assessment, Development, and Evaluation (GRADE) approach. This involved a five-step process as outlined by Goldet and Howick [42], resulting in a final rating of the quality of evidence as high, moderate, low, or very low. Based on GRADE guidelines, evidence from observational studies was initially rated as low quality, but could be upgraded in the presence of specific factors such as large effect sizes or clear dose–response relationships. Cohen’s *d* values were used to estimate effect sizes. Conversely, the certainty was downgraded based on concerns related to risk of bias, inconsistency, imprecision, indirectness, or potential publication bias. Publication bias was assessed by generating funnel plots using the funnel function from the “metafor” package (v. 4.6.0) in R. Due to the small number of studies in our meta-analysis (fewer than 10), formal statistical tests such as Egger’s regression test were not performed as they lack sufficient statistical power with small sample sizes and can lead to misleading results [43]. Instead, we relied on visual inspection of the funnel plot to qualitatively evaluate potential asymmetry that might suggest publication bias. The GRADE approach was applied to the studies included in the meta-analysis, following the removal of the outlier (described below), to enable a tailored interpretation of the evidence in accordance with established guidelines for evaluating observational studies [44].

## Results

### Study selection

**Fig. 1:**
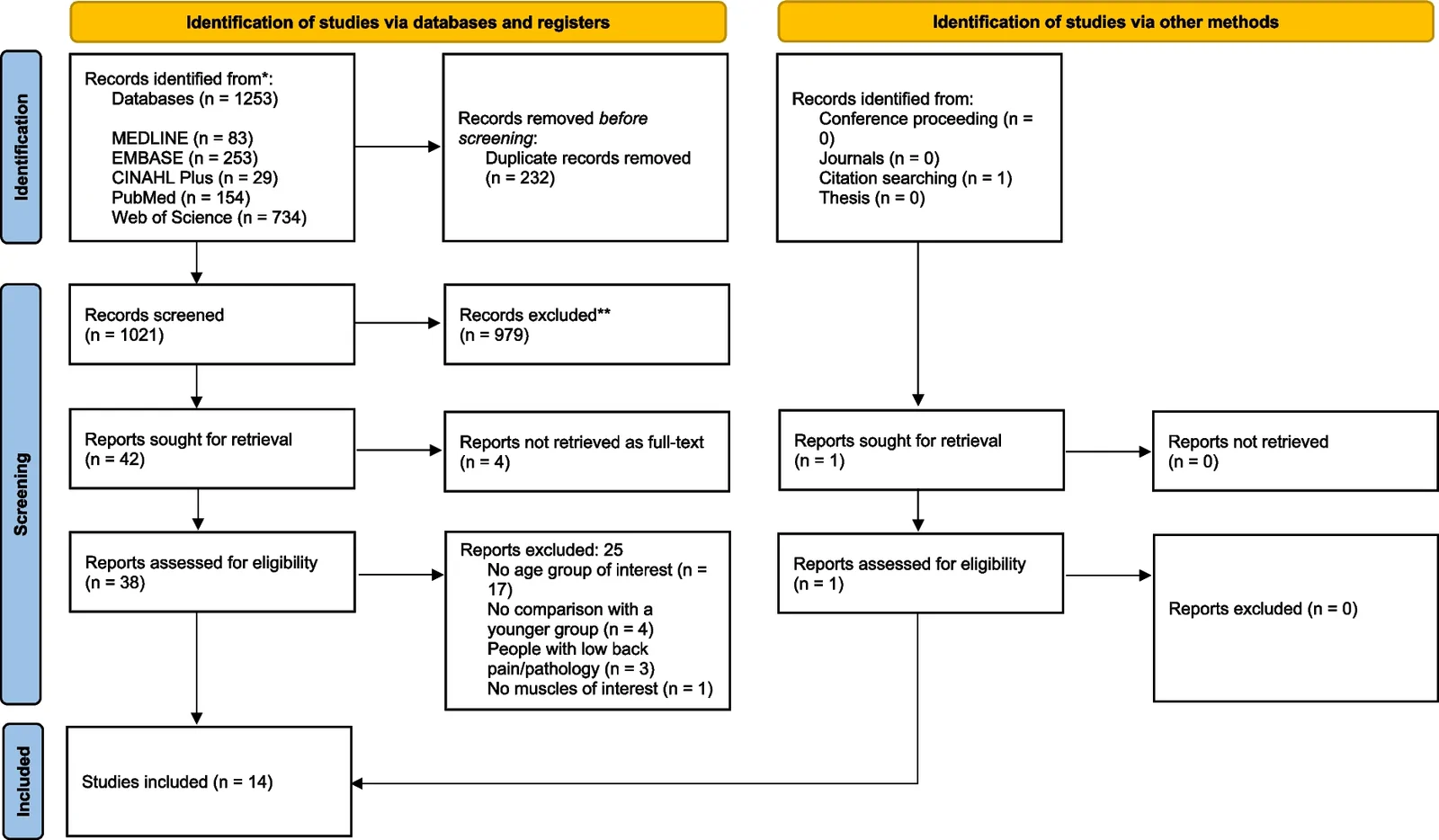
PRISMA flow diagram

The database search process yielded a total of 1253 records. Following duplicate removal, two independent reviewers screened the titles and abstracts of 1021 records. An additional paper was identified through hand-searching. Full-text screening was performed on 43 records in total (42 from databases and 1 from hand-searching). Four studies were excluded from this phase as the full text was not available: three were conference abstracts [45–47], and requests for the full text of one study were unsuccessful [48]. During the full-text screening, studies were excluded for the following reasons: 17 did not include the age groups of interest (i.e., older group < 60 years) [49–65]; four lacked comparisons with younger groups [66–69]; three involved individuals with low back pain or other medical conditions [70–72]; one did not assess the muscles of interest [73]. The review ultimately included 14 studies (13 from databases and 1 from hand-searching). The complete screening process is illustrated in the PRISMA flow diagram in Fig. 1 [25].

### Study characteristics

Although 14 studies were initially included in this review, the study by Parreira et al. (2014) [19] was excluded due to suspected data overlap with a prior publication from the same authors [74]. This resulted in a final inclusion of 13 studies, the characteristics of which are summarised in Table 1. In total, these studies involved 1116 participants, of whom 326 were older individuals, while the remaining were young and middle-aged controls. All studies focused on fatigue/endurance of the back extensor muscles, while no studies investigating the neck extensor muscles in the context of fatigue and ageing were retrieved. When specific muscles were identified in the studies (i.e., in those assessing EMG), their names were explicitly reported in Table 1 (e.g., multifidus, lumbar erector spinae). In contrast, when studies evaluated back muscles more generally, these muscles were described using the broader term “paraspinal muscles”. Additionally, all studies employed isometric tasks, except for Parreira et al. [74], which used a dynamic fatiguing protocol.

**Table 1:** Characteristics of the 13 included studies

Study	Elderly characteristics	Control group characteristics	Muscle/s assessed	Fatiguing task	Measurement tool	Outcome measures	Results
Koch et al. (2025)	**10 elderly** (65.0 ± 5.0 years, 5 M, 5 F, 24.7 ± 5.8 kg/m^2^)	**10 young** (25.6 ± 2.0 years, 5 M, 5 F, 22.3 ± 2.3 kg/m^2^)	**Paraspinal muscles**	**Modified Sørensen test on a 45° Roman Chair until voluntary exhaustion** (the trunk was maintained unsupported on the Roman Chair in a horizontal position relative to the ground)	**/**	**Endurance time**	**Endurance time results:** - Elderly: 222 ± 98 s - Young: 368 ± 154 s Mean ± SD values *No statistical comparison was provided, as endurance time was a secondary outcome in the study. Therefore, only descriptive values are reported
Parrella et al. (2025)	**13 elderly** (69 ± 4 years, 8 M, 5 F, 172 ± 8 cm, 70.23 ± 8.17 kg, 23.58 ± 1.80 kg/m^2^) Mean ± SD values	**13 young** (26 ± 2 years, 8 M, 5 F, 173 ± 13 cm, 70.62 ± 15.19 kg, 23.32 ± 2.44 kg/m^2^) Mean ± SD values	**Lumbar erector spinae muscle**	**Modified Sørensen test on a 45° Roman Chair at 30% maximal voluntary isometric force (MVIF) until voluntary exhaustion** (the trunk was maintained unsupported on the Roman Chair in a horizontal position relative to the ground)	**HD-sEMG placed on the right erector spinae** (64-electrode grid from L5 to L2 approximately)	**- Endurance time** **- Spatial distribution of muscle activity (x and y coordinates of the centroid of the topographical map)** at 0%, 25%, 50%, 75% and 100% of endurance time **- MFCV** at 0%, 25%, 50%, 75% and 100% of endurance time **- MVIF decline (%)** after the task	**No significant differences in endurance time** between the two age groups (*p* = 0.749): - Elderly: 99.74 ± 34.03 s - Young: 104.32 ± 38.17 s **Significant age-related differences for centroid displacement along both the x** (*F* = 6.043, η_p_^2^ = 0.201, *p* = 0.006) **and y axis** (*F* = 5.111, η_p_^2^ = 0.176, *p* = 0.017) **over time**: - Elderly: no significant changes along the x axis (*p* > 0.05), but cranial shift of the centroid from 0 to 25% (*p* = 0.003), 0% to 75% (*p* = 0.003), 0% to 100% (*p* = 0.004), 50% to 75% (*p* = 0.002), and from 50 to 100% (*p* = 0.006); - Young: lateral displacement of the centroid between 0 and 100% (*p* = 0.004), but no changes along the y-axis (*p* > 0.05) **No differences in muscle fibre conduction velocity decline between groups** (*F* = 0.177, η_p_^2^ = 0.007, *p* = 0.752) **No differences in MVIF decline between the two groups** (*p* = 0.681): - Elderly: −22.70 ± 17.67% - Young: −20.45 ± 8.2% Mean ± SD values
Han et al. (2024)	**59 elderly** (65.95 ± 4.48 years, 29 M, 30 F, 164.71 ± 8.65 cm, 66.44 ± 9.22 kg, 24.46 ± 2.53 kg/m^2^); - **Physical activity index (PAI)**: 25.52 ± 8.11 Mean ± SD values	**29 young** (27.41 ± 4.32 years, 14 M, 15 F, 169.69 ± 7.79 cm, 66 ± 15.34 kg, 22.65 ± 3.59 kg/m^2^); - **PAI**: 39.58 ± 5.45; **93 middle-aged** (51.85 ± 4.72 years, 43 M, 50 F, 164.23 ± 8.69 cm, 68.11 ± 13.69 kg, 25.17 ± 4.41 kg/m^2^); - smoking (yes): 22; - **PAI**: 30.38 ± 6.65 Mean ± SD values	**Paraspinal muscles**	**Ito test** performed until task failure or for a maximum of 5 min (prone position on examination table with a pad under the lower abdomen; trunk raised ~15° with arms parallel to the body, cervical spine neutral, and feet maintained on the table)	**/**	**Endurance time**	**↓ Endurance time in older adults** compared to both young and middle-aged subjects (*p* < 0.001): - Elderly: 120.07 ± 78 s - Middle-aged: 162.22 ± 73.77 s - Young: 182.97 ± 51.78 s **↓ Physical activity level in elderly** compared to both young and middle-aged subjects (*p* < 0.001) Mean ± SD values
Liu et al. (2024)	**18 elderly** (65.17 ± 4.72 years, 24.08 ± 1.92 kg/m^2^) Mean ± SD values	**27 young** (29.44 ± 5.71 years, 21.00 ± 2.98 kg/m^2^); **49 middle-aged** (49.73 ± 4.57 years, 22.76 ± 2.11 kg/m^2^) Mean ± SD values	**Paraspinal muscles**	**Ito test** performed until task failure (prone position on examination table with a pad under the lower abdomen; trunk raised ~15° with arms parallel to the body, cervical spine neutral, and feet maintained on the table)	**/**	**Endurance time**	**No significant differences in endurance time** between the three groups (*p* = 0.135): - Elderly: 36.22 ± 30.69 s - Middle-aged: 53.74 ± 43.99 s - Young: 63.72 ± 46.04 s Mean ± SD values
Da Silva et al. (2015)	**10 elderly** (73 ± 7 years, 160 ± 10 cm, 67 ± 10 kg, 26 ± 4 kg/m^2^) Mean ± SD values	**10 young** (30 ± 4 years, 165 ± 12 cm, 64 ± 8 kg, 23 ± 2 kg/m^2^) Mean ± SD values	**Multifidus** and** iliocostalis lumborum muscles**	2 different isometric fatiguing protocols: - **45° Roman chair exercise** with the trunk maintained unsupported in a horizontal position relative to the ground **for 60 s;** - **Functional task** consisting of maintaining a box, equivalent to 10% of body mass, close to the trunk in the upright position **for 60 s**	**8 sEMG bipolar electrodes placed bilaterally on the multifidus** at L5 level and **iliocostalis** at L3 level	**- Normalised EMG index of muscle fatigue** (NMFslp) defined as the decline in the MF over time - **BORG CR-10 scale** throughout the fatiguing tasks	**No significant differences in NMFslp** and **BORG scale values** between age groups during the **Roman chair exercise**: - Multifidus (*p* = 0.143): −0.17 ± 0.18%/s in elderly, −0.17 ± 0.23%/s in young - Iliocostalis (*p* = 0.079): −0.11 ± 0.16 in elderly, −0.25 ± 0.15%/s in young - Borg (*p* = 0.206) **No significant differences in NMFslp** and **BORG scale values** between age groups during the **functional task**: - Multifidus (*p* = 0.689): −0.10 ± 0.14%/s in elderly, −0.12 ± 0.29%/s in young - Iliocostalis (*p* = 0.217): −0.10 ± 0.23%/s in elderly, −0.20 ± 0.24%/s in young - Borg (*p* = 0.380) Results from each muscle are averaged between the two side (mean ± SD values)
Kienbacher et al. (2014)	**42 elderly** (21 F, 67.11 ± 1.55 years, 25.15 ± 0.45 kg/m^2^) - **IPAQ score**: 424.09 ± 59.07 Mean ± SE values	**44 young** (19 F, 33.14 ± 1.66 years, 24.11 ± 0.47 kg/m^2^) - **IPAQ score**: 337.15 ± 60.25 Mean ± SE values	**Multifidus, longissimus and iliocostalis lumborum muscles**	**Sustained isometric back extension test at 80% of MVC for 30s** (participants were seated on the device with the trunk flexed forward at 30°)	**6 double parallel-bar sEMG electrodes placed bilaterally on the multifidus** at L5 level, **longissimus** at L2 level and **iliocostalis** at L1 level	**Absolute and normalised MF slope declines** over the task	**↓ Absolute MF slope decline in elderly at L5 level only** (−0.14 ± 0.03 Hz/s in elderly, −0.24 ± 0.04 in young; *F* = 04.26, *p* = 0.04) **↓ Normalised MF slope decline in elderly at L5 level only** (−0.12 ± 0.03%/s in elderly, −0.26 ± 0.04%/s in young; *F* = 06.40, *p* = 0.01) **No differences in physical activity level between age groups** (*p* > 0.05) Mean ± SE values
Tsuboi et al. (2013)	**- 11 elderly men** (71.1 ± 3.6 years, 168 ± 4.8 cm, 67.0 ± 7.1 kg, 23.8 ± 2.1 kg/m^2^) **- 11 elderly women** (67.6 ± 3.3 years, 153 ± 3.7 cm, 53.9 ± 7.1 kg, 22.9 ± 3.0 kg/m^2^) Mean ± SD values	**- 17 young men** (25.1 ± 3.2 years, 172 ± 3.9 cm, 63.2 ± 5.5 kg, 21.4 ± 1.8 kg/m^2^) **- 14 young women** (25.6 ± 1.8 years, 158 ± 5.3 cm, 51.5 ± 4.7kg, 20.7 ± 1.3 kg/m^2^) Mean ± SD values	**Lumbar erector spinae muscle**	**Biering-Sørensen back endurance test until voluntary exhaustion** (prone on a bed with the anterior superior iliac spines at the edge; trunk maintained in a horizontal position relative to the lower body with hands on the head)	**One active sEMG electrode on the left erector spinae** at L1 level (with one reference electrode on L1 spinous process)	**- Slope of MF values** and **MPF values** over the task **- Endurance time**	**Elderly men:** **- ↓ MF slopes** compared to young men (−21.6 ± 11.8% per min in elderly, −29.3 ± 8.1% per min in young; *p* < 0.05) **- ↓ MPF slopes** compared to young men (−15.6 ± 6.4% per min in elderly, −24.5 ± 7.4% per min in young; *p* < 0.01) **- no differences in endurance time** (95 ± 44 s in elderly, 98 ± 21 s in young; *p* > 0.05) **Elderly women:** **- no differences in MF slopes** compared to young women (−17.1 ± 7.9% per min in elderly, 19.9 ± 5.5% per min in young; *p* > 0.05) **- no differences in MPF slopes** compared to young women (−12.8 ± 4.9% per min in elderly, 11.1 ± 4.5% per min in young; *p* > 0.05) **- no differences in endurance time** compared to young women (129 ± 59 s in elderly, 143 ± 48 s in young; *p* > 0.05) Mean ± SD values
Parreira et al. (2013)	**18 elderly** (69.8 ± 4.9 years, 9 M, 157 ± 7 cm, 65.9 ± 9 kg, 26.5 ± 3.1 kg/m^2^) Mean ± SD values	**18 young** (26.3 ± 5.8 years, 9 M, 168 ± 10 cm, 64.6 ± 13.3 kg, 22.4 ± 2.6 kg/m^2^) Mean ± SD values	**Paraspinal muscles**	**Dynamic back extension exercise on a 45° Roman Chair until voluntary exhaustion** (2 s of flexion and 2 s of extension from 45° to 0° relative to the ground at 60/bpm)	**/**	**Endurance time**	**↑ Endurance time in elderly** compared to young (*p* = 0.039): - Elderly: 133 ± 52 s - Young: 97 ± 27 s Mean ± SD values
Bašič et al. (2013)	**14 elderly men** (72.0 ± 7.2 years, 172 ± 6 cm, 77.9 ± 13.5 kg) Mean ± SD values	**16 young men** (27.5 ± 4.1 years, 178 ± 5 cm, 74.5 ± 8.1 kg). Mean ± SD values	**Paraspinal muscles**	**Modified Sørensen test on a 45° Roman Chair until voluntary exhaustion** (the trunk was maintained unsupported on the Roman Chair in a horizontal position relative to the ground)	**/**	**Endurance time**	**No differences in endurance time** between elderly and young participants (*p* = 0.82): - Elderly: 3.1 ± 1.8 min - Young: 4.2 ± 1.2 min Mean ± SD values
Adedoyin et al. (2011)	**Group aged 60 + (*N* = *31*)** (62.8 ± 3.0 years, 165 ± 10 cm, 69.2 ± 11.7 kg, 25.1 ± 4.4 kg/m^2^) Mean ± SD values	**- Group aged 19–29 (*N* = *210*)** (23.2 ± 2.4 years, 168 ± 10 cm, 63.3 ± 9.7 kg, 22.4 ± 3.2 kg/m^2^) **- Group aged 30–39 (*N* = *103*)** (33.4 ± 2.9 years, 166 ± 10 cm, 64.5 ± 11.7 kg, 23.3 ± 4.1 kg/m^2^) **- Group aged 40–49 (*N* = *128*)** (43.4 ± 3.0 years, 165 ± 10 cm, 66.9 ± 13.7 kg, 24.2 ± 4.5 kg/m^2^) **- Group aged 50–59 (*N* = *89*)** (53.8 ± 2.6 years, 164 ± 10 cm, 66.7 ± 12.2 kg, 24.8 ± 4.6 kg/m^2^) Mean ± SD values	**Paraspinal muscles**	**Biering-Sørensen back endurance test until voluntary exhaustion** (prone on a bed with the anterior superior iliac spines at the edge; trunk maintained in a horizontal position relative to the lower body with arms parallel to the body)	**/**	**Endurance time**	**↓ Endurance time with increasing age** (*F* = 32.702; *p* = 0.001) - Group aged 19–29: 133 ± 41 s - Group aged 30–39: 121 ± 49 s - Group aged 40–49: 103 ± 42 s - Group aged 50–59: 82 ± 36 s - Group aged 60 + : 81 ± 33 s Endurance time differed significantly between paired groups (*p* < 0.05), except between age groups 50–59 and 60 + years (*p* > 0.05) Mean ± SD values
Mbada et al. (2011)	**Group aged 60–69 (*N* = *47*)** (62.0 ± 2.56 years, 165 ± 7 cm, 67.9 ± 11.4 kg, 24.9 ± 4.13 kg/m^2^) Mean ± SD values	**- Group aged 20–29 (*N* = *336*)** (23.7 ± 2.32 years, 167 ± 9 cm, 62.6 ± 10.2 kg, 22.4 ± 3.42 kg/m^2^) **- Group aged 30–39 (*N* = *143*)** (33.3 ± 2.92 years, 166 ± 9 cm, 63.3 ± 11.0 kg, 23.0 ± 3.94 kg/m^2^) **- Group aged 40–49 (*N* = *199*)** (43.4 ± 3.05 years, 165 ± 9 cm, 66.6 ± 13.2 kg, 24.4 ± 4.61 kg/m^2^) **- Group aged 50–59 (*N* = *199*)** (53.8 ± 2.55 years, 164 ± 9 cm, 66.6 ± 13.2 kg, 24.7 ± 4.71 kg/m^2^) Mean ± SD values	**Paraspinal muscles**	**Biering-Sørensen back endurance test until voluntary exhaustion** (prone on a bed with the anterior superior iliac spines at the edge; trunk maintained in a horizontal position relative to the lower body with arms parallel to the body)	**/**	**Endurance time**	**↓ Endurance time with increasing age** (*F* = 54.197; *p* = 0.001) - Group aged 11–19: 137.0 ± 64.8 s - Group aged 20–29: 138.4 ± 42.6 s - Group aged 30–39: 121.9 ± 47.8 s - Group aged 40–49: 102.7 ± 41.2 s - Group aged 50–59: 81.8 ± 36.2 s - Group aged 60–69: 77.9 ± 31.3 s Endurance time differed significantly between paired groups (*p* < 0.05) Mean ± SD values
Singh et al. (2011)	**26 elderly** (72.1 ± 5.9 years, 10 M, 16 F) Mean ± SD values	**26 young** (27.9 ± 5.2 years, 10 M, 16 F) Mean ± SD values	**Longissimus muscle**	**Isometric lumbar extension contraction at 60% MVC for 120 s** (participants stood upright facing a load cell connected at the 12th thoracic level by a metal cable in a restraining frame with the pelvis and legs immobilised)	**2 bipolar EMG electrodes placed bilaterally on the longissimus muscle** at L3 level	**Power of the EMG signals** in 5 different frequency bands (20–100 Hz; 101–200 Hz; 201–300 Hz; 301–400 Hz and 401–499 Hz) at 1 s, 40 s, 80 s and 120 s of task duration	**↑ Power of the EMG signals in the 101–200 Hz band in elderly** compared with younger adults in all time epochs (p < 0.05): - 1 s: 29.46 ± 4.23% in elderly, 25 ± 5.10% in young - 40 s: 29.82 ± 4.09% in elderly, 25.44 ± 4.45% in young - 80 s: 29.16 ± 3.8% in elderly, 25.15 ± 4.31% in young - 120 s: 29.89 ± 3.22% in elderly, 25 ± 4.16% in young Results are averaged between the two sides Mean ± SD values
Champagne et al. (2009)	**16 elderly men** (72.8 ± 4.7 years, 171 ± 10 cm, 79 ± 6.7 kg, 27.1 ± 2.6 kg/m^2^) Mean ± SD values	**20 young men** (22.8 ± 3.1 years, 1.79 ± 10 cm, 77.3 ± 10.8 kg, 24.1 ± 2.5 kg/m^2^) Mean ± SD values	**Paraspinal muscles**	**Modified Sørensen test on a 45° Roman Chair until voluntary exhaustion** (the trunk was maintained unsupported on the Roman Chair in a horizontal position relative to the ground)	**/**	**- Endurance time** **- Decline in maximal voluntary isometric lift force (%)** after the fatiguing task **- Borg CR-10 scale** measured throughout the task at successive 10% intervals of total endurance time - **Time to exhaustion at Borg-7 rate** (expressed as % of total endurance time)	**No differences in endurance time between groups** (296.3 ± 84.1 s in elderly, 334.9 ± 80.4 s in young; *p* = 0.17) **No differences in maximal isometric lift force decline between groups** (10.3% ± 9.0% in elderly; 5.9% ± 9.2% in young; *T* = −1.433; *p* = 0.16) **No between-group differences in Borg scale** throughout the fatiguing task (*F* = 2.62; *p* = 0.11) - 10%: 1.7 ± 0.26 in elderly, 1.51 ± 0.23 in young - 20%: 2.26 ± 0.27 in elderly, 2.07 ± 0.29 in young - 30%: 3.26 ± 0.26 in elderly, 2.87 ± 0.27 in young - 40%: 4.62 ± 0.36 in elderly, 3.76 ± 0.2 in young - 50%: 5.85 ± 0.37 in elderly, 4.89 ± 0.35 in young - 60%: 7.03 ± 0.44 in elderly, 6.21 ± 0.35 in young - 70%: 8.04 ± 0.39 in elderly, 7.33 ± 0.34 in young - 80%: 9.02 ± 0.28 in elderly, 8.54 ± 0.25 in young - 90%: 9.64 ± 0.22 in elderly, 9.31 ± 0.15 in young - 100%: 10 in elderly, 10 in young **No differences between age groups in the time remaining to exhaustion at Borg-7 rate** (43.40 ± 3.1% in elderly; 38.58 ± 2.61% in young; *T* = −1.22; *p* = 0.23) Mean ± SEM values for force decline, Borg and time at Borg-7 rate Mean ± SD values for endurance time

### Risk of bias assessment

A comprehensive summary of the risk of bias for each study is presented in Table 2. Scores ranged from 10 to 17 out of a maximum of 18, corresponding to percentages between 55.56% and 94.44%. None of the included studies has been rated as “poor” quality study. The full set of questions, along with the specific scores assigned to each question for each study, is presented in Supplemental file 3.

**Table 2:**
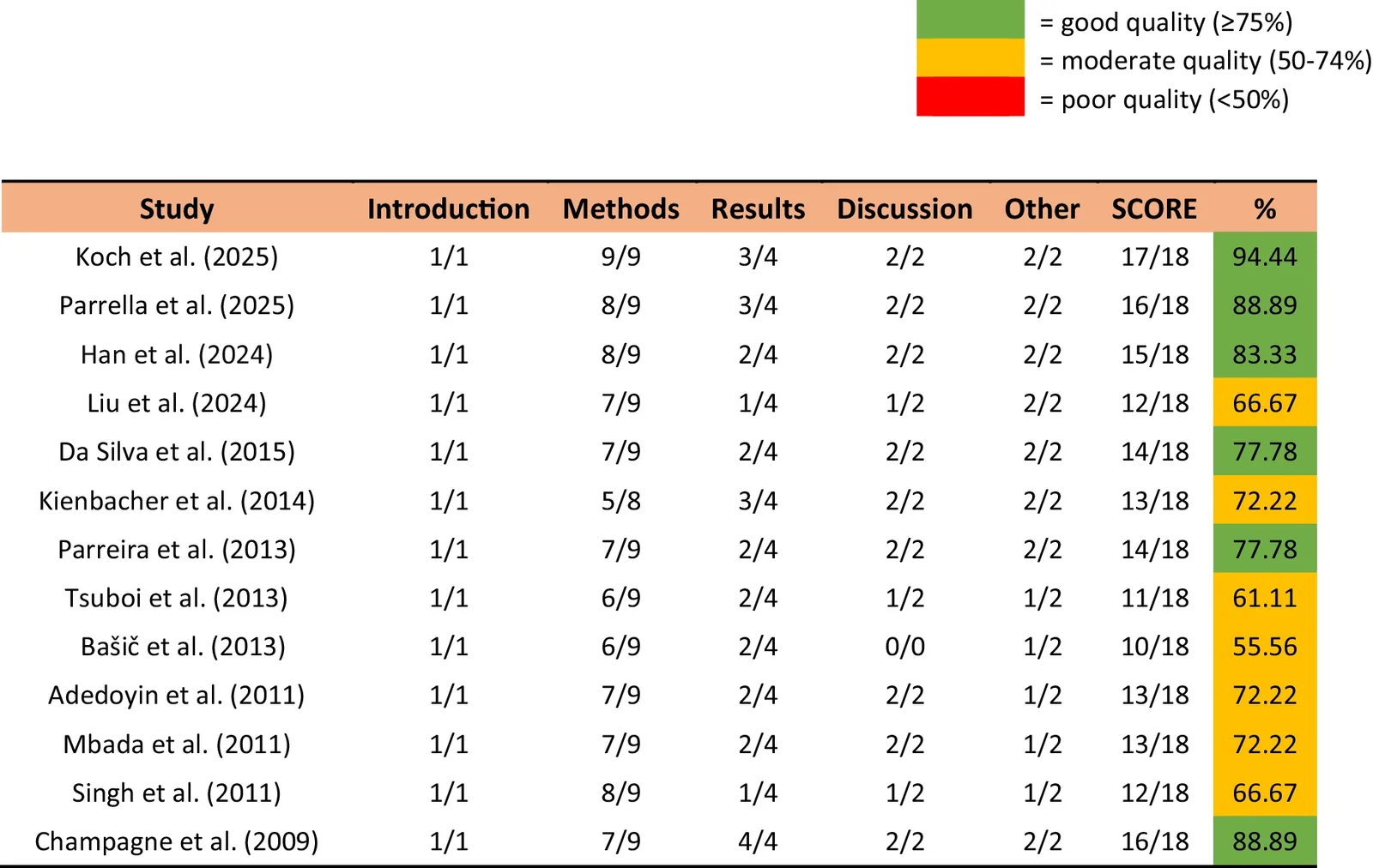
Risk of bias assessment of the included studies using the AXIS

### Narrative synthesis of the results

The main findings from the individual studies were grouped per outcome domains and described in a narrative way. Ten studies assessed endurance time [17, 18, 20, 21, 24, 74–78], five studies evaluated EMG parameters [22–24, 78, 79], and two studies investigated force decline [21, 78].

#### Endurance time

Three studies reported reduced endurance time in older compared to younger individuals, specifically during the Biering-Sørensen endurance test [18, 76] and the Ito test [17]. In contrast, five studies found no significant differences in endurance time between younger and older participants [20, 21, 24, 75, 78]. Three of these studies employed a modified version of the Sørensen test performed on a 45° Roman Chair until voluntary exhaustion [20, 21, 78], with Parrella et al. [78] using a submaximal intensity set at 30% of maximal voluntary isometric force (MVIF). Instead, the other two studies used the Ito test [75] and the standard Biering-Sørensen test [24]. In the study by Koch et al. [77], which employed a modified version of the Sørensen test, endurance time was reported only as a secondary outcome, and no statistical comparison between age groups was conducted. Therefore, no specific findings are discussed in the narrative synthesis; however, the descriptive data (Table 1) were included in the meta-analysis. Lastly, one paper reported greater endurance time in older individuals compared to younger controls during a dynamic back extension task until voluntary exhaustion [74].

#### EMG parameters

Three studies assessed the decline in MF slope during fatiguing tasks for the back extensors [22–24], with Tsuboi et al. [24] also evaluating the decline in MPF. One study found a reduced decline in both absolute and normalised MF values in older adults compared to younger controls for the erector spinae assessed at the L5 level during a 30-s isometric back extension task at 80% MVC [22]. Similarly, Tsuboi et al. [24] reported lower MF and MPF slope declines in the lumbar erector spinae of older men during the Biering-Sørensen test performed until voluntary exhaustion, although no such differences were observed in older women. Additionally, no differences in MF decline between age groups were observed in the multifidus and iliocostalis lumborum muscles by da Silva et al. [23] during either a 60-s 45° Roman chair exercise or a functional task involving holding a box in an upright position for 60 s. In contrast, Singh et al. [79] found that older adults exhibited higher EMG signal power in the 101–200 Hz frequency band of the longissimus muscle compared to younger adults during all time epochs of a 120-s isometric lumbar extension task performed at 60% MVC. Lastly, Parrella et al. [78], using high-density electromyography (HD-sEMG), found comparable declines in MFCV of the lumbar erector spinae muscle between age groups during a modified version of the Biering-Sørensen test performed at 30% MVIF to exhaustion. However, age-related differences in the spatial distribution of muscle activity were observed, with older adults exhibiting a cranial shift in the centroid of muscle activity with fatigue, while younger individuals showed a lateral displacement.

#### Force decline

One study assessed the decline in maximal isometric lift force following a modified version of the Sørensen test performed until voluntary exhaustion [21] and found no differences between older and younger individuals. Similarly, Parrella et al. [78] observed a comparable reduction in MVIF across age groups using the same test, but performed at a submaximal intensity (30% MVIF).

### Meta-analysis results for endurance time

#### Initial analysis including all studies

A random-effects meta-analysis was initially conducted including all 10 identified studies, comprising 958 participants (248 elderly and 710 young adults). This preliminary analysis indicated that the elderly exhibited significantly reduced endurance of the back extensor muscles compared to younger adults (MD = −33.91 s [95% CI: −62.55; −5.26], t_9_ = −2.68, *p* = 0.025; Supplemental file 4). However, substantial heterogeneity was observed (I^2^ = 87.0%, τ^2^ = 1091.87, χ^2^ = 69.27, *p* < 0.001), warranting further investigation of potential influential studies.

#### Identification of outliers

Influence diagnostics were performed to identify potential outliers that might disproportionately affect the meta-analysis results. This quantitative assessment clearly identified Parreira et al. (2013) (study 5) as a significant outlier (Supplemental file 5). This finding was consistent across several diagnostic measures, particularly evident in the Studentised Residuals, DFFITS values, Cook’s Distances, Covariance ratios, estimates of heterogeneity (τ^2^ and Q-statistic), and DFBETAS plots. The specific values from the influence diagnostics analysis are provided in Supplemental file 6. The methodological assessment confirmed this statistical identification, as Parreira et al. (2013) was the only study assessing trunk fatigue during a dynamic task, whereas all other studies evaluated trunk muscle endurance during a sustained back extension isometric contraction. Additionally, it was the only study showing significantly better endurance in older adults compared to younger adults (MD = 36.00 [95% CI: 8.93; 63.07]), contrary to the pattern observed across all other included studies. Due to these statistical and methodological considerations, we determined that Parreira et al. (2013) should be excluded from the primary analysis.

#### Refined analysis after outlier removal

**Fig. 2:**
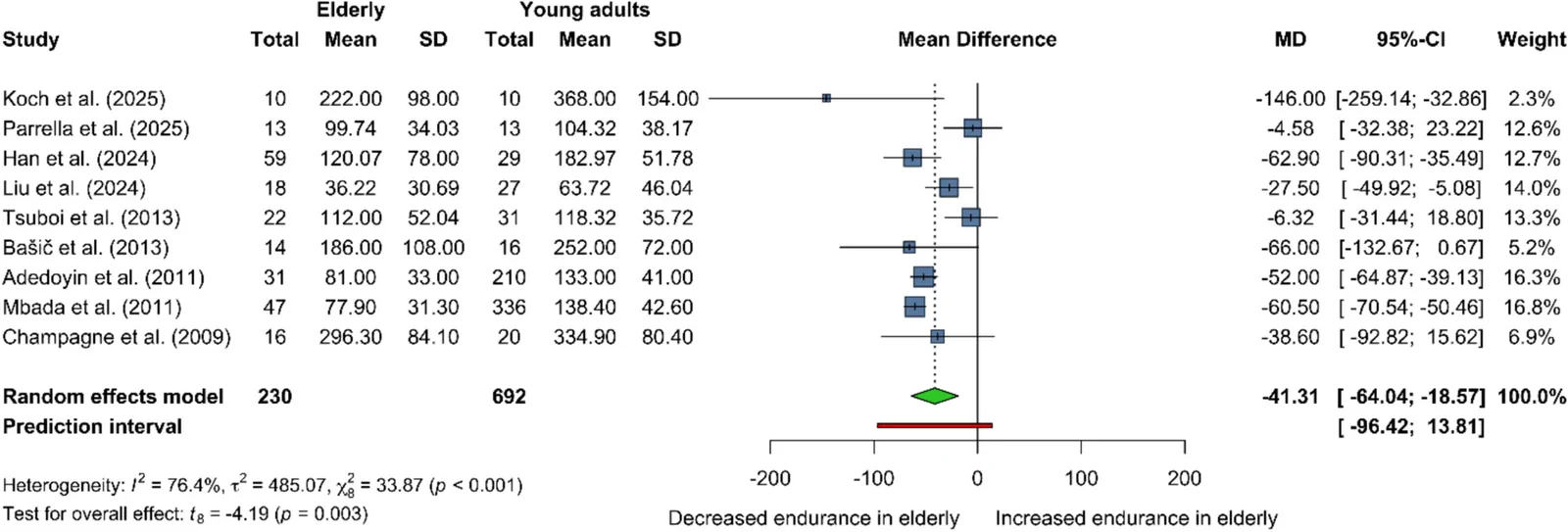
Forest plot of endurance time differences between elderly and young adults after outlier removal. The plot of the refined analysis displays mean differences (MD) in endurance time (seconds) with corresponding 95% confidence intervals (95% CI) for each included study. The green diamond represents the pooled effect estimate with its 95% CI, while the red line indicates the prediction interval. Negative values on the x-axis indicate decreased endurance time in elderly compared to young adults, while positive values indicate increased endurance in elderly adults. The size of each square is proportional to the study’s weight in the meta-analysis. The forest plot is organized in descending order based on publication year

After excluding Parreira et al. (2013), our refined meta-analysis included 9 studies with a total of 922 participants (230 elderly and 692 young adults). The results demonstrated a larger and more statistically significant effect, with older adults exhibiting markedly reduced endurance times of the back extensor muscles compared to younger adults (MD = −41.31 [95% CI: −64.04; −18.57], t_8_ = −4.19, *p* = 0.003; Fig. 2). Heterogeneity, although still present, was substantially reduced (I^2^ = 76.4%, τ^2^ = 485.07).

**Fig. 3:**
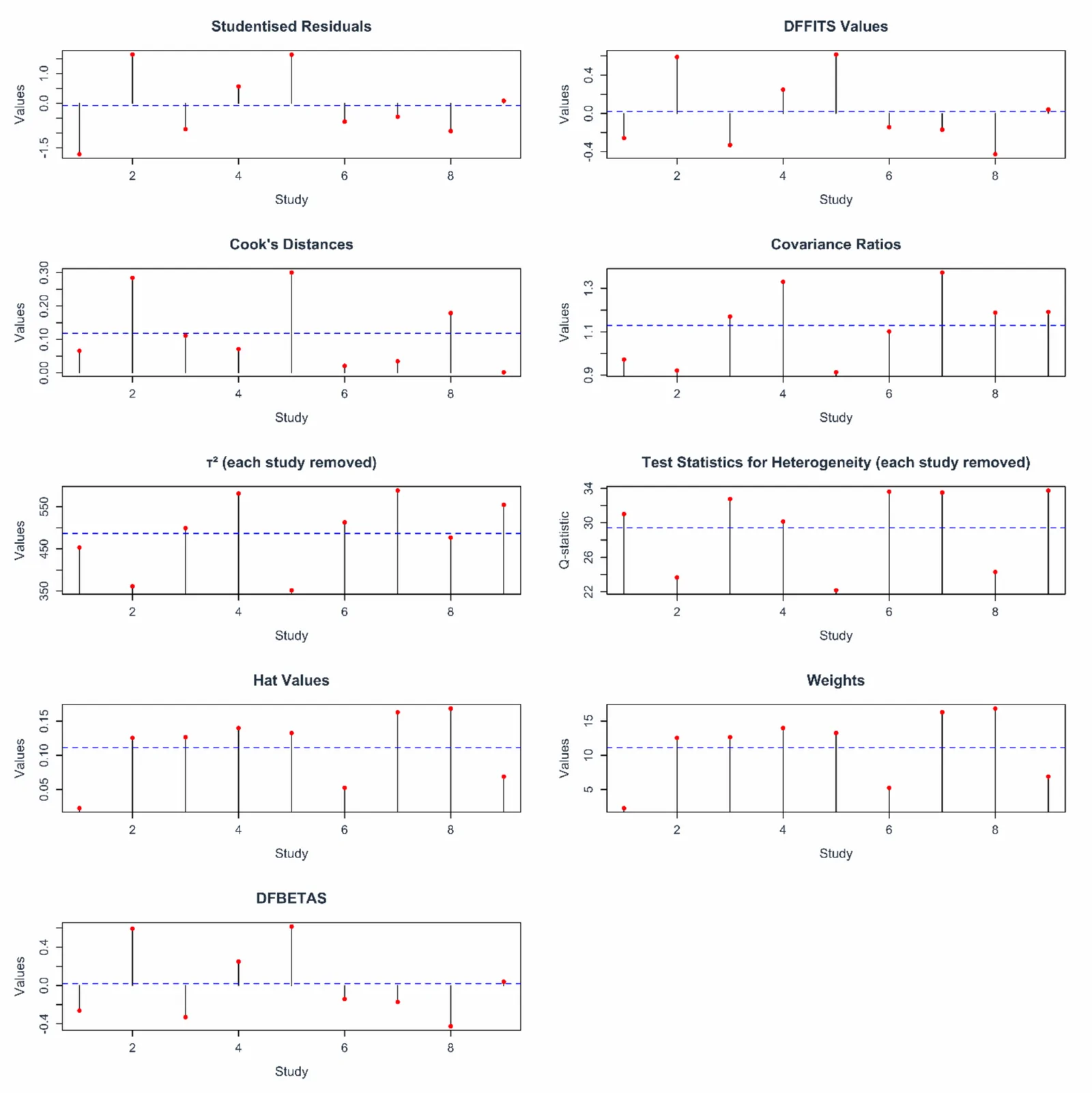
Influence diagnostic plots for the meta-analysis of endurance time differences between elderly and young adults. The figure presents an influence analysis for the meta-analytic model, illustrating various diagnostic measures to identify influential studies. The plots include externally studentised residuals, DFFITS values, Cook’s distances, covariance ratios, estimates of heterogeneity (τ^2^ and Q-statistic) when each study is removed, hat values, weights and DFBETAS. The y-axis shows the values of each measure, and the x-axis displays the study numbers, ordered according to their appearance in the forest plot. The blue line in each plot represents the mean of each parameter across all studies

**Fig. 4:**
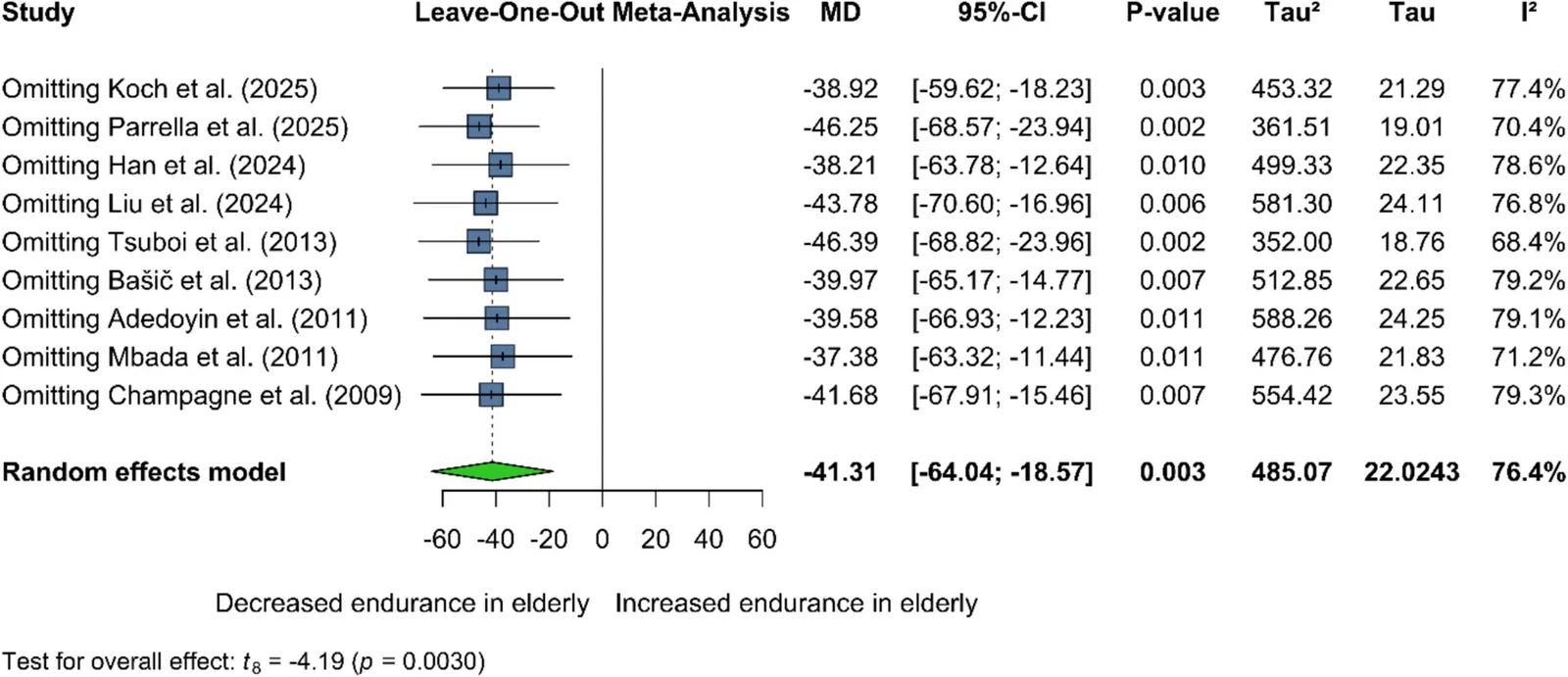
Forest plot showing sensitivity analysis with sequential removal of individual studies after exclusion of Parreira et al. (2013). Mean differences (MD) with 95% confidence intervals are shown for the meta-analysis after sequentially omitting each remaining study, following the exclusion of Parreira et al. (2013). The green diamond at the bottom represents the overall effect from the analysis. Each row shows how the pooled effect estimates, confidence intervals, and heterogeneity statistics (*P*-value, Tau^2^, Tau, and I^2^) change when the corresponding study is removed

Secondary influence diagnostics performed on the refined dataset did not identify any studies as potential outliers (Fig. 3). The specific values from the influence diagnostics analysis are provided in Supplemental file 7. Sensitivity analyses confirmed the robustness of our findings, as the effect sizes remained significant across all iterations regardless of which study was removed from the model (Fig. 4).

#### Assessment of publication bias

**Fig. 5:**
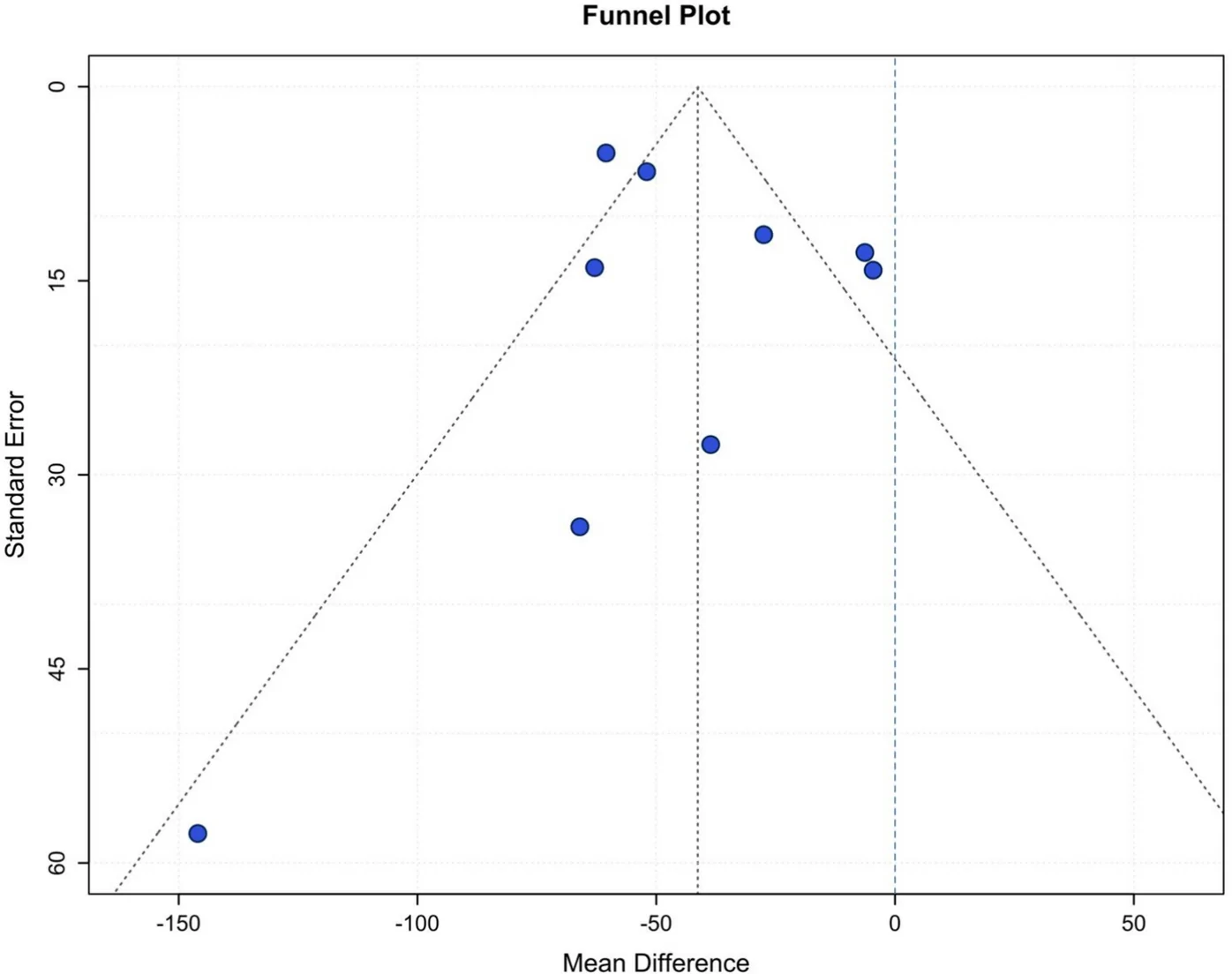
Funnel plot for the assessment of publication bias. The blue dots represent individual studies, with mean difference (seconds) on the x-axis and standard error on the y-axis. The vertical black dashed line indicates the overall effect

The funnel plot (Fig. 5) shows a relatively symmetrical distribution of studies around the central effect estimate, with studies appearing on both sides of the mean effect. While there is some clustering of studies on the left side (negative effects), this is consistent with the overall negative effect observed in the meta-analysis rather than indicative of bias. The reasonable symmetry of the plot suggests that publication bias is unlikely to substantially influence our findings, although caution is warranted given the small number of included studies.

#### GRADE

Considering all the domains assessed using the GRADE approach, the findings from the meta-analysis provide moderate certainty of evidence that the endurance time of the back extensor muscles during isometric tasks is reduced with ageing. The included studies were not downgraded for risk of bias or publication bias as the AXIS tool and the funnel plot did not reveal any major concerns, respectively. For inconsistency, no downgrade was needed despite high heterogeneity (*I*^2^ = 76.4%) because the direction of the effect was consistent across studies. Therefore, the substantial statistical heterogeneity likely reflects methodological differences between studies, such as variations in sample size, rather than true inconsistency in the effect estimates. Additionally, indirectness was not a concern given that the outcome variable (endurance time) was consistently measured across all studies, and the target population was appropriately represented. No downgrade was applied for imprecision because the confidence interval did not cross the line of no effect (zero), with both upper and lower bounds indicating a meaningful difference. Lastly, an upgrade was applied as the calculated Cohen’s *d* for our outcome indicated a medium to large effect size (–0.77). The summary of the results is presented in Table 3.

**Table 3:** Summary of the overall certainty of evidence

Study	Outcome	Rating	Reasons for downgrading (↓) or upgrading (↑)
Koch et al. (2025) Parrella et al. (2025) Han et al. (2024) Liu et al. (2024) Tsuboi et al. (2013) Bašič et al. (2013) Adedoyin et al. (2011) Mbada et al. (2011) Champagne et al. (2009)	Reduced endurance with ageing during isometric back extension tasks	**○○ ⊕ ○** MODERATE certainty of evidence	**↑** Medium to large Cohen’s *d* (−0.77)

## Discussion

The aim of this systematic review and meta-analysis was to synthesise the existing evidence on how ageing influences the development of muscle fatigue in the spinal extensor muscles, including the lumbar, thoracic, and cervical (neck) extensors. However, only findings related to the back extensor muscles are reported and discussed, as no studies were identified that examined and compared fatigue in the neck extensors between younger and older adults. Based on data from 9 studies, a meta-analysis revealed, with moderate certainty of evidence, a significant reduction in endurance time of the back extensor muscles during sustained isometric contractions in older adults. In contrast, EMG studies investigating neuromuscular fatigue in the back extensor muscles produced mixed findings, likely due to considerable methodological variability across studies.

### Is endurance of the back extensor muscles reduced in older adults?

Across all studies included in the meta-analysis, the observed effect consistently suggested a decline in endurance time with ageing (Fig. 2). The substantial heterogeneity among studies is likely attributable to differences in participant characteristics (e.g., sample size). Additionally, there may be an overlap in the populations reported by two studies [18, 76], as their characteristics appear very similar, but one study involves a larger sample size. Attempts to clarify this potential overlap with the authors were unsuccessful. Nevertheless, sensitivity analysis confirmed the robustness of the findings, as the exclusion of any single study did not alter the overall outcome.

Contrary to our findings, a previous systematic review by Christie et al. [80] reported a general increased fatigue resistance in elderly people compared to young adults during isometric contractions of lower and upper limb muscles. This phenomenon, defined as the “fatigue paradox”, has been attributed to physiological changes associated with ageing, such as greater loss and atrophy of fast-twitch muscle fibres, motor unit remodeling, and a reduced reliance on glycolytic metabolism [81]. Altogether, these factors contribute to slower contractile properties in older adults. However, this mechanism may not apply as prominently to the back extensor muscles (e.g., the erector spinae), which already exhibit a high proportion of slow-twitch fibres in younger individuals (approximately 60–70%) [82]. Therefore, although age-related physiological changes likely occur in these muscles as well, the functional consequences may not be as evident as in muscles with a greater proportion of fast-twitch fibres, such as the elbow flexors and knee extensors.

The studies included in the meta-analysis employed various types of isometric tests to assess the endurance capacity of the back extensor muscles, including the standard Biering-Sørensen test [18, 24, 76], a modified version of the Biering-Sørensen test using a 45° Roman chair [20, 21, 77, 78], and the Ito test [17, 75]. These protocols have been widely used in the literature to evaluate back muscle function across diverse populations [29]. Liu et al. [75] observed a positive correlation between the functional cross-sectional area (FCSA) of the multifidus muscle and static back muscle endurance. In contrast, other studies did not report any significant associations between the CSA of the paraspinal muscles and endurance time [17, 68, 83]. However, there is consistent evidence indicating a negative correlation between fat infiltration within the paraspinal muscles and back endurance [17, 75, 83]. Therefore, since elderly people exhibit greater fat infiltration in both the multifidus and erector spinae muscles compared to younger individuals [17, 75], this factor may partly explain the reduced endurance time of the back extensors observed for older adults.

Notably, the three studies reporting the greatest reductions in endurance time among elderly individuals (Fig. 2) employed different isometric fatiguing protocols: the standard Biering-Sørensen test [76], a modified Sørensen test on a 45° Roman Chair [20, 77], and the Ito test [17]. Although these protocols impose varying mechanical demands on the back extensor muscles [29], the reduction in endurance time observed in older participants was consistently evident across tests. This finding suggests that the observed age-related decline in back extensor endurance is not attributable to the specific characteristics of a single test, but rather reflects a broader, more generalisable physiological decline associated with ageing. Nevertheless, with the exception of Parrella et al. [78], none of the studies included in the meta-analysis quantified the force output during the endurance task, which was generally performed at an absolute intensity rather than relative to individual capacity. This methodological aspect may have influenced the magnitude of the age-related difference in endurance time, since tasks performed at an absolute force level may impose a higher relative workload on older adults due to their reduced maximal strength. However, even when controlling for force output—as in Parrella et al. [78], who employed a task performed at 30% of participants’ maximal voluntary isometric force—the forest plot (Fig. 2) still indicates a trend toward reduced endurance time with ageing. Additionally, studies that assessed general fatigue indicators, such as post-task force decline [21, 78] and perceived exertion using the Borg scale [21], reported no significant age-related differences in these parameters, suggesting that overall fatigue development was comparable between older and younger individuals. This is further supported by da Silva et al. [23] who observed no group differences in Borg scores during two distinct submaximal fatiguing tasks not performed to exhaustion. Although only a few studies assessed these parameters, the findings suggest that the reduced endurance time observed in older adults might not be related to a greater general sense of fatigue, but rather to specific neuromuscular changes associated with ageing.

### Potential neuromuscular mechanisms

From a neuromuscular perspective, some studies examined EMG parameters during fatiguing tasks for the back extensor muscles to better understand the mechanisms underlying age-related fatigue. A smaller decline in MF was reported in older adults during a 30-s fatiguing task at 80% MVC [22]. Similarly, a reduced decline in both MF and mean MPF was observed in older men during the Biering-Sørensen test until voluntary exhaustion [24]. These attenuated spectral shifts have been interpreted as potentially reflecting a greater proportion of type I muscle fibres with ageing. However, Tsuboi et al. [24] did not observe a corresponding increase in back muscle endurance, unlike findings reported in other muscle groups [84]. This discrepancy suggests that factors beyond muscle fibre-type composition may primarily underlie the age-related changes in EMG spectral shift, such as increased reliance on synergistic muscles. Older adults may recruit additional synergistic muscles more extensively than younger individuals to help sustain the endurance task, distributing the workload more broadly and consequently producing smaller EMG spectral shifts in the primary back extensor muscles. Moreover, Parrella et al. [78] did not find significant differences in MFCV between age groups during a modified Biering-Sørensen test, further suggesting that intrinsic muscle fibre properties may not fully explain the attenuated spectral shifts observed in older adults. Taken together, these findings point toward alternative explanations, such as greater recruitment of synergistic muscles or altered load-sharing strategies in older individuals to maintain task performance. In this regard, older adults exhibited a different distribution of erector spinae muscle activity compared to young controls during fatigue. Specifically, they showed a more cranial activation pattern, which may reflect a compensatory strategy to redistribute load away from the lower lumbar segments—particularly the L4–L5 level—which are more susceptible to age-related degenerative changes [85]. In contrast, no differences in MF decline were reported during either a 60-s 45° Roman chair exercise or a functional task involving holding a box in an upright position for 60 s [23], possibly due to the lower intensity and shorter duration of these tasks compared to more demanding protocols employed in the other studies. Lastly, Singh et al. (2011) [79] reported greater power in the 101–200 Hz frequency band of EMG signals in older adults compared to younger individuals during a 120-s isometric lumbar extension contraction at 60% MVC. However, no time-related changes were observed in any frequency band for either age group, leading the authors to suggest that this type of task likely did not induce significant fatigue-related changes in neuromuscular activation patterns. Overall, the variability in EMG outcomes and methodological approaches across studies limits the ability to draw definitive conclusions about the neuromuscular mechanisms underlying age-related fatigue of the back extensor muscles. Furthermore, to the best of the authors’ knowledge, no animal studies have specifically assessed age-related changes in endurance or fatigue of these muscles, and therefore mechanistic insights from animal models cannot currently be applied to human back extensor fatigue.

### Methodological considerations

Some important considerations should be made when interpreting age-related differences in muscle fatigue, which may guide future research in this area. First, future studies should extend beyond isometric protocols to include dynamic tasks involving the spinal extensors, as age-related fatigue patterns may differ depending on the type of contraction [84]. For instance, the assessment of isokinetic contractions of the spinal extensor muscles may provide a more comprehensive understanding of how endurance capacity of these muscles changes with ageing. This is particularly relevant as such contractions more closely reflect the movements performed during activities of daily living compared with isometric contractions [86]. Notably, the only study focusing on dynamic contractions of the back extensor muscles [74] reported greater endurance in elderly individuals compared to young participants, contrasting with the reduced endurance observed during isometric tasks in this review. However, future studies are needed to confirm or refute this finding. Additionally, physical activity levels should be assessed, as they may influence not only muscular endurance capacity but also an individual’s predisposition to sustain prolonged effort. Among the included studies, only Han et al. [17] and Kienbacher et al. [22] quantified physical activity levels in their respective age groups. Lastly, sex differences in spinal extensor muscle fatigue should also be investigated, as previous research suggests that sex can be a factor influencing fatigue resistance during ageing [87], but it remains unclear whether similar patterns extend to spinal muscles. Reporting both physical activity levels and sex differences is important as these variables could serve as potential moderating factors in future meta-analyses. 

In conclusion, this systematic review and meta-analysis revealed that endurance of the back extensor muscles is reduced in older adults when performing isometric tasks. However, the specific neuromuscular mechanisms underlying this decline remain unclear due to mixed EMG findings and methodological variability across studies. Future studies should aim to clarify these mechanisms, potentially by employing protocols based on relative intensity (e.g., %MVC) rather than absolute loads and should also extend to dynamic contractions. Moreover, studies specifically examining age-related fatigue in the neck extensor muscles are warranted, as no such data were identified in the present review.

## Supplementary Information

Below is the link to the electronic supplementary material.

ESM 1 (DOCX 33.2 KB)

ESM 2 (DOCX 21.3 KB)

ESM 3 (XLSX 16.1 KB)

ESM 4 (DOCX 235 KB)

ESM 5 (DOCX 283 KB)

ESM 6 (XLSX 10.6 KB)

ESM 7 (XLSX 10.3 KB)

## Data Availability

The template data collection forms, data extracted from included studies, data used for all analyses, analytic code and any other materials used in the review are available upon reasonable request. Interested researchers can contact the lead author, Martina Parrella, for access to these materials.

## References

[1] Das S. ‘A comparative study to know the effectiveness of prone back extension exercises and swiss ball exercises on back extensor muscles performance’. *International Journal of Physiotherapy*. 2016;3. 10.15621/ijphy/2016/v3i4/111052

[2] Ghamkhar L, Kahlaee AH. The effect of trunk muscle fatigue on postural control of upright stance: a systematic review. Gait Posture. 2019;72:167–74. 31207565 10.1016/j.gaitpost.2019.06.010

[3] Gandevia SC. Spinal and supraspinal factors in human muscle fatigue. Physiol Rev. 2001. 10.1152/physrev.2001.81.4.1725. 11581501

[4] Lin D, Nussbaum MA, Seol H, et al. Acute effects of localized muscle fatigue on postural control and patterns of recovery during upright stance: influence of fatigue location and age. Eur J Appl Physiol. 2009;106:425–34. 10.1007/s00421-009-1026-5. 19306019

[5] Johanson E, Brumagne S, Janssens L, et al. The effect of acute back muscle fatigue on postural control strategy in people with and without recurrent low back pain. Eur Spine J. 2011;20:2152–9. 10.1007/s00586-011-1825-3. 21533851 PMC3229729

[6] Granata KP, Gottipati P. Fatigue influences the dynamic stability of the torso. Ergonomics. 2008;51(8):1258–71. 10.1080/00140130802030722. 18608477

[7] Enthoven P, Skargren E, Kjellman G, et al. Course of back pain in primary care: a prospective study of physical measures. J Rehabil Med. 2003;35:168–73. 10.1080/16501970310013517. 12892242

[8] Reddy RS, Maiya AG, Rao SK. Effect of dorsal neck muscle fatigue on cervicocephalic kinaesthetic sensibility. Hong Kong Physiother J. 2012;30:105–9. 10.1016/j.hkpj.2012.06.002.

[9] Reddy RS, Meziat-Filho N, Ferreira AS, et al. Comparison of neck extensor muscle endurance and cervical proprioception between asymptomatic individuals and patients with chronic neck pain: neck muscle endurance and proprioception. J Bodyw Mov Ther. 2021;26:180–6. 10.1016/j.jbmt.2020.12.040. 33992242

[10] Dallaway A, Kite C, Griffen C, et al. Age-related degeneration of the lumbar paravertebral muscles: systematic review and three-level meta-regression. Exp Gerontol. 2020. 10.1016/j.exger.2020.110856. 32006635

[11] Okada E, Matsumoto M, Ichihara D, et al. Cross-sectional area of posterior extensor muscles of the cervical spine in asymptomatic subjects: a 10-year longitudinal magnetic resonance imaging study. Eur Spine J. 2011;20:1567–73. 10.1007/s00586-011-1774-x. 21431426 PMC3175907

[12] Beauchamp MK, Jette AM, Ni P, et al. Leg and trunk impairments predict participation in life roles in older adults: results from Boston RISE. J Gerontol A Biol Sci Med Sci. 2016;71:663–9. 10.1093/gerona/glv157. 26369668 PMC5007738

[13] Suri P, Kiely DK, Leveille SG, et al. Increased trunk extension endurance is associated with meaningful improvement in balance among older adults with mobility problems. Arch Phys Med Rehabil. 2011;92:1038–43. 10.1016/j.apmr.2010.12.044. 21636073 PMC3124606

[14] Safiri S, Kolahi AA, Hoy D, *et al.* Global, regional, and national burden of neck pain in the general population, 1990–2017: Systematic analysis of the Global Burden of Disease Study 2017. *The BMJ*. 2020;368. 10.1136/bmj.m791 PMC7249252 32217608

[15] Teichert F, Karner V, Döding R, et al. Effectiveness of exercise interventions for preventing neck pain: a systematic review with meta-analysis of randomized controlled trials. J Orthop Sports Phys Ther. 2023;53:594–609. 37683100 10.2519/jospt.2023.12063

[16] Cheng C, su hao-T, Yen L-W, *et al.* Long-term effects of therapeutic exercise on nonspecific chronic neck pain: a literature review. 2015. 10.1589/jpts.27.1271 PMC4434025 25995604

[17] Han G, Wang W, Yue L, et al. Age-dependent differences of paraspinal muscle endurance and morphology in Chinese community population without chronic low back pain. Global Spine J. 2024;14:235–43. 10.1177/21925682221103507. 35584688 PMC10676164

[18] Adedoyin RA, Mbada CE, Farotimi AO, et al. Endurance of low back musculature: normative data for adults. J Back Musculoskelet Rehabil. 2011;24:101–9. 10.3233/BMR-2011-0282. 21558615

[19] Parreira RB, De Oliveira MR, Amorim CF, et al. Older adults present better back endurance than young adults during a dynamic trunk extension exercise. J Back Musculoskelet Rehabil. 2014;27:153–9. 10.3233/BMR-130430. 23963267

[20] Bašič D, Strojnik V, Rugelj D. The effect of back muscle fatigue on postural sway. Kinesiologia Slovenica : scientific journal on sport - ISSN. 2013;1318–2269(19):5–16.

[21] Champagne A, Descarreaux M, Lafond D. Comparison between elderly and young males’ lumbopelvic extensor muscle endurance assessed during a clinical isometric back extension test. J Manipulative Physiol Ther. 2009;32:521–6. 10.1016/j.jmpt.2009.08.008. 19748403

[22] Kienbacher T, Habenicht R, Starek C, et al. The potential use of spectral electromyographic fatigue as a screening and outcome monitoring tool of sarcopenic back muscle alterations. J Neuroeng Rehabil. 2014. 10.1186/1743-0003-11-106. 24985941 PMC4105890

[23] da Silva RA, Vieira ER, Cabrera M, et al. Back muscle fatigue of younger and older adults with and without chronic low back pain using two protocols: a case-control study. J Electromyogr Kinesiol. 2015;25:928–36. 10.1016/j.jelekin.2015.10.003. 26542483

[24] Tsuboi H, Nishimura Y, Sakata T. Age-related sex differences in erector spinae muscle endurance using surface electromyographic power spectral analysis in healthy humans. Spine J. 2013;13:1928–33. 10.1016/j.spinee.2013.06.060. 24021621

[25] Page MJ, McKenzie JE, Bossuyt PM, et al. The PRISMA 2020 statement: an updated guideline for reporting systematic reviews. Syst Rev. 2021. 10.1186/s13643-021-01626-4. 33781348 PMC8008539

[26] Devecchi V, Rushton AB, Gallina A, et al. Are neuromuscular adaptations present in people with recurrent spinal pain during a period of remission? A systematic review. PLoS ONE. 2021. 10.1371/journal.pone.0249220. 33793608 PMC8016280

[27] Arvanitidis M, Falla D, Sanderson A, et al. Does pain influence control of muscle force? A systematic review and meta-analysis. Eur J Pain. 2024. 10.1002/ejp.4716. 39176440 PMC11671343

[28] UNHCR. UNHCR definition of older persons. 2025. 17/07/2025 https://emergency.unhcr.org/protection/persons-risk/older-persons/ (accessed 17 July 2025)

[29] Demoulin C, Vanderthommen M, Duysens C, et al. Spinal muscle evaluation using the Sorensen test: a critical appraisal of the literature. Joint Bone Spine. 2006;73:43–50. 16461206 10.1016/j.jbspin.2004.08.002

[30] Drevon D, Fursa SR, Malcolm AL. Intercoder reliability and validity of WebPlotDigitizer in extracting graphed data. Behav Modif. 2017;41:323–39. 10.1177/0145445516673998. 27760807

[31] Downes MJ, Brennan ML, Williams HC, *et al.* Development of a critical appraisal tool to assess the quality of cross-sectional studies (AXIS). *BMJ Open*. 2016;6. 10.1136/bmjopen-2016 PMC5168618 27932337

[32] McArthur K, Jorgensen D, Climstein M, et al. Epidemiology of acute injuries in surfing: type, location, mechanism, severity, and incidence: a systematic review. Sports. 2020. 10.3390/sports8020025. 32093200 PMC7076752

[33] Lunt E, Ong T, Gordon AL, et al. The clinical usefulness of muscle mass and strength measures in older people: a systematic review. Age Ageing. 2021;50:88–95. 32706848 10.1093/ageing/afaa123 PMC7793605

[34] McKenzie JE, Brennan SE, Ryan RE, *et al.* Summarizing study characteristics and preparing for synthesis. *Cochrane handbook for systematic reviews of interventions. Wiley-Blackwell*. 2019:229–40.

[35] Deeks JJ, Higgins JP, Altman DG, *et al.* Analysing data and undertaking meta-analyses. *Cochrane handbook for systematic reviews of inter ventions. Wiley-Blackwell*. 2019:241–84.

[36] R Core Team. *R: a language and environment for statistical computing. R Foundation for Statistical Computing*. 2022.

[37] Balduzzi S, Rücker G, Schwarzer G. How to perform a meta-analysis with R: a practical tutorial. Evid Based Ment Health. 2019;22:153–60. 10.1136/ebmental-2019-300117. 31563865 PMC10231495

[38] Jackson D, Law M, Rücker G, et al. The Hartung-Knapp modification for random-effects meta-analysis: a useful refinement but are there any residual concerns? Stat Med. 2017;36:3923–34. 10.1002/sim.7411. 28748567 PMC5628734

[39] Inthout J, Ioannidis JPA, Rovers MM, et al. Plea for routinely presenting prediction intervals in meta-analysis. BMJ Open. 2016;6:10247. 10.1136/bmjopen-2015. PMC4947751 27406637

[40] Viechtbauer W. Conducting meta-analyses in R with the metafor package. 2010.

[41] Viechtbauer W, Cheung MWL. Outlier and influence diagnostics for meta-analysis. Res Synth Methods. 2010;1(2):112–25. 10.1002/jrsm.11. 26061377

[42] Goldet G, Howick J. Understanding GRADE: an introduction. J Evid Based Med. 2013;6:50–4. 10.1111/jebm.12018. 23557528

[43] Sterne JAC, Egger M. Funnel plots for detecting bias in meta-analysis: guidelines on choice of axis. 2001. 10.1016/s0895-4356(01)00377-8 11576817

[44] Mueller M, D’Addario M, Egger M, et al. Methods to systematically review and meta-analyse observational studies: a systematic scoping review of recommendations. BMC Med Res Methodol. 2018. 10.1186/s12874-018-0495-9. 29783954 PMC5963098

[45] Piovanelli B, Gobbo M, Donzelli S, *et al.* Paravertebral muscles metabolism changes with age in agreement with EMG particularly at L4–5 : a Near Infrared Spectroscopy evaluation (NIRS). 2019.

[46] Ebenbichler GR, Kollmitzer J, Mair P, et al. Spectral electromyographic fatigue as a potential screening and outcome monitoring tool of sarcopenic back muscle alterations. PM&R. 2014;9:6. 10.1186/1743-0003-11-106 PMC4105890 24985941

[47] Sagendorf KS, Manini TM, Mayer JM, et al. Relationship of strength and endurance of the low back between younger and older subjects. Med Sci Sports Exerc. 2000;32:S243.

[48] Tsuboi H, Tanina H, Yasuoka Y, et al. Spectral electromyographic fatigue analysis of erector spinae muscle in prepubertal girls compared with young adult and elderly women. Physiotherapy. 2015;1:101.

[49] Habenicht R, Ebenbichler G, Bonato P, et al. Age-specific differences in the time-frequency representation of surface electromyographic data recorded during a submaximal cyclic back extension exercise: a promising biomarker to detect early signs of sarcopenia. J Neuroeng Rehabil. 2020. 10.1186/s12984-020-0645-2. 31992323 PMC6986160

[50] Kell RT, Bhambhani Y. Relationship between erector spinae muscle oxygenation via in vivo near infrared spectroscopy and static endurance time in healthy males. Eur J Appl Physiol. 2008;102:243–50. 10.1007/s00421-007-0577-6. 17968582

[51] Sibson BE, Harris AR, Yegian AK, et al. Associations of back muscle endurance with occupational back muscle activity and spinal loading among subsistence farmers and office workers in Rwanda. PLoS ONE. 2024;19:e0309658. 10.1371/journal.pone.0309658. 39495738 PMC11534227

[52] Park J-W, Yumin K, Park S. The effect of proprioceptive position sense by fatigue of low back muscles. J Korean Soc Phys Ther. 2012;2012(24):414–8.

[53] Vlazna D, Adamova B, Krkoska P, et al. Strength and endurance of the lumbar extensor muscles and their predictors: a cross-sectional study in healthy subjects. J Electromyogr Kinesiol. 2025. 10.1016/j.jelekin.2024.102973. 39765107

[54] Kurz E, Anders C, Walther M, et al. Force capacity of back extensor muscles in healthy males: effects of age and recovery time. J Appl Biomech. 2014;30:713–21. 10.1123/jab.2013-0308. 25102100

[55] Valkeinen H, Ylinen J, Mälkiä E, et al. Maximal force, force/time and activation/coactivation characteristics of the neck muscles in extension and flexion in healthy men and women at different ages. Eur J Appl Physiol. 2002;88:247–54. 10.1007/s00421-002-0709-y. 12458368

[56] Latikka P, Batti MC, DMSci Tv, *et al.* Correlations of isokinetic and psychophysical back lift and static back extensor endurance tests in men. 1995. 10.1016/0268-0033(94)00003-p 11415575

[57] van Dieën JH, Westebring-van der Putten EP, Kingma I, *et al.* Low-level activity of the trunk extensor muscles causes electromyographic manifestations of fatigue in absence of decreased oxygenation. *Journal of Electromyography and Kinesiology*. 2009;19:398–406. 10.1016/j.jelekin.2007.11.010 18178450

[58] Peolsson A, Hamp C, Albinsson AK, et al. Test position and reliability in measurements of dorsal neck muscle endurance. Adv Physiother. 2007;9:181–9. 10.1080/14038190701702058.

[59] Lariviere C, Gravel D, Gagnon D, et al. Toward the development of predictive equations of back muscle capacity based on frequency- and temporal-domain electromyographic indices computed from intermittent static contractions. Spine J. 2009;9:87–95. 18082457 10.1016/j.spinee.2007.09.012

[60] Kankaanpaa M, Luaksonen D, Airaksinen SO, et al. Age, sex, and body mass index as determinants of back and hip extensor fatigue in the isometric sfirensen back endurance test. Arch Phys Med Rehabil. 1998;79:1069–75. 9749686 10.1016/s0003-9993(98)90173-3

[61] Hiepe P, Gussew A, Rzanny R, et al. Age-related structural and functional changes of low back muscles. Exp Gerontol. 2015;65:23–34. 10.1016/j.exger.2015.02.016. 25735850

[62] Yassierli, Nussbaum MA. Effects of age, gender, and task parameters on fatigue development during intermittent isokinetic torso extensions. *Int J Ind Ergon*. 2009;39:185–91. 10.1016/j.ergon.2008.05.003

[63] Yassierli, Nussbaum MA, Iridiastadi H, *et al.* The influence of age on isometric endurance and fatigue is muscle dependent: a study of shoulder abduction and torso extension. *Ergonomics*. 2007;50:26–45. 10.1080/00140130600967323 17178650

[64] Ebenbichler GR, Unterlerchner L, Habenicht R. Estimating neural control from concentric vs. eccentric surface electromyographic representations during fatiguing, cyclic submaximal back extension exercises. Front Physiol. 2017;8:299. 28559851 10.3389/fphys.2017.00299 PMC5432577

[65] Chen C, Zhang C, Tang Y, et al. Quantitative assessments of paraspinal muscles and their relationship with lumbar extensor muscle function based on Dixon magnetic resonance imaging techniques. J Back Musculoskelet Rehabil. 2025. 10.1177/10538127251321769. 40183424

[66] Ropponen A, Levalahti E, Videman T, *et al.* The role of genetics and environment in lifting force and isometric trunk extensor endurance. *Phys Ther*. 2004;608–21. 15225080

[67] Keshavarzi F, Azadinia F, Talebian S, et al. Impairments in trunk muscles performance and proprioception in older adults with hyperkyphosis. J Man Manip Ther. 2022;30:249–57. 10.1080/10669817.2022.2034403. 35133255 PMC9344955

[68] Gibbons L, Latikka P, Videman T, et al. The association of trunk muscle cross-sectional area and magnetic resonance image parameters with isokinetic and psychophysical lifting strength and static back muscle endurance in men. J Spinal Disord. 1997;10:398–403. 9355056

[69] Laura Gibbons. *Back function testing and paraspinal muscle magnetic resonance image parameters: their associations and determinants: a study on male, monozygotic twins*. 1998.

[70] Troup J, Chapman AE. Changes in the waveform of the electro myogram during fatiguing activity in the muscles of the spine and hips the analysis of postural stress. Electromyogr Clin Neurophysiol. 1972;12:347–65. 4645621

[71] Chen C, Yang S, Tang Y, et al. Correlation between strength/endurance of paraspinal muscles and sagittal parameters in patients with degenerative spinal deformity. BMC Musculoskelet Disord. 2023. 10.1186/s12891-023-06747-6. 37563700 PMC10413613

[72] Ebenbichler GR, Habenicht R, Ziegelbecker S, et al. Age- and sex-specific effects in paravertebral surface electromyographic back extensor muscle fatigue in chronic low back pain. Geroscience. 2020;42(1):251–69. 10.1007/s11357-019-00134-7. 31773454 PMC7031171

[73] Lesniewski L-A, Sinning WE. Relationship between strength and muscle fatigue in older adults during a 30-second chair stand test. Med Sci Sports Exerc. 2000;32:S277–S277.

[74] Parreira RB, Amorim CF, Gil AW. Effect of trunk extensor fatigue on the postural balance of elderly and young adults during unipodal task. Eur J Appl Physiol. 2013;113:1989–96. 10.1007/s00421-013-2627-6. 23543068

[75] Liu Y, Yuan L, Zeng Y, et al. Relationship between paraspinal muscle morphology and function in different directions in a healthy Chinese population at different ages: a cross-sectional study. BMC Musculoskelet Disord. 2024. 10.1186/s12891-024-07842-y. 39277728 PMC11401413

[76] Mbada CE, Ayanniyi O, Adedoyin RA, et al. Back muscles’ endurance in adolescents and adults: normative data for a sub-Saharan African population. J Musculoskelet Res. 2011. 10.1142/S0218957711500047.

[77] Koch D, Nüesch C, Ignasiak D, et al. Age and activity but not lumbar spinal stenosis and muscle fatigue affect sagittal spinal alignment: a pilot study. Clin Biomech. 2025. 10.1016/j.clinbiomech.2025.106577. 40472708

[78] Parrella M, Borzuola R, Siciliano FP, et al. Fatigue-induced alterations in the spatial distribution of lumbar erector spinae activity in older versus young adults. Eur J Appl Physiol. 2025. 10.1007/s00421-025-05864-5. 40571745

[79] Singh DKA, Bailey M, Lee R. Strength and fatigue of lumbar extensor muscles in older adults. Muscle Nerve. 2011;44:74–9. 10.1002/mus.21998. 21488056

[80] Christie A, Snook EM, Kent-Braun JA. Systematic review and meta-analysis of skeletal muscle fatigue in old age. Med Sci Sports Exerc. 2011;43:568–77. 10.1249/MSS.0b013e3181f9b1c4. 20881888 PMC3705929

[81] Hunter SK, Pereira HM, Keenan KG. The aging neuromuscular system and motor performance. J Appl Physiol. 2016;121:982–95. 10.1152/japplphysiol.00475.2016. 27516536 PMC5142309

[82] Jørgensen K, Nicholaisen T, Kato M. Muscle fiber distribution, capillary density, and enzymatic activities in the lumbar paravertebral muscles of young men. Significance for isometric endurance. Spine (Phila Pa 1976). 1993;18:1439–50. 8235814

[83] Yazici A, Yerlikaya T. Investigation of the relationship between the clinical evaluation results of lumbar region muscles with cross-sectional area and fat infiltration. J Back Musculoskelet Rehabil. 2022;35:1277–87. 10.3233/BMR-210241. 35599467

[84] Avin KG, Law LAF. Age-related differences in muscle fatigue vary by contraction type: a meta-analysis. Phys Ther. 2011;91:1153–65. 10.2522/ptj.20100333. 21616932 PMC3145894

[85] Hicks GE, Morone N, Weiner DK. Degenerative lumbar disc and facet disease in older adults: prevalence and clinical correlates. Spine (Phila Pa 1976). 2009;34:1301–6. 10.1097/BRS.0b013e3181a18263. 19455005 PMC2867597

[86] Paris MT, McNeil CJ, Power GA, et al. Age-related performance fatigability: a comprehensive review of dynamic tasks. J Appl Physiol. 2022;133:850–66. 35952347 10.1152/japplphysiol.00319.2022

[87] Allman BL, Rice CL. Neuromuscular fatigue and aging: central and peripheral factors. Muscle Nerve. 2002;25:785–96. 12115966 10.1002/mus.10116

