# Supplementary material for: The effects of ageing on fatigue and endurance of the spinal extensor muscles: a systematic review and meta-analysis
Source: GeroScience. 2025 Nov 8;48(4):5785–809. doi: 10.1007/s11357-025-01987-x (PMC13575018; doi:10.1007/s11357-025-01987-x)
Supplement: Supplementary file 2 [file 11357_2025_1987_MOESM2_ESM.docx]

**Article title**: The effects of ageing on fatigue and endurance of the spinal extensor muscles: A systematic review and meta-analysis

**Journal name**: GeroScience

**Authors**: Martina Parrella^1^, Michail Arvanitidis^2^, Andrea Macaluso^1^, Deborah Falla^2^

**Affiliations**: ^1^Department of Movement, Human and Health Sciences, Università degli Studi di Roma “Foro Italico”, Rome, Italy.

^2^Centre of Precision Rehabilitation for Spinal Pain (CPR Spine), School of Sport, Exercise and Rehabilitation Sciences, College of Life and Environmental Sciences, University of Birmingham, Birmingham, UK.

**Corresponding author e-mail**: Professor Deborah Falla ([*d.falla@bham.ac.uk*](mailto:d.falla@bham.ac.uk))

- **SEARCH STRATEGY** (Medline, EMBASE, PubMed, Web of Science, CINAHL)

**MEDLINE / EMBASE**

1) Spine/

2) Spinal muscles.mp

3) Back Muscles/

4) Paraspinal Muscles/

5) Erector spinae.mp.

6) Multifidus.mp.

7) Trunk extensor muscles.mp.

8) Lumbar extensor muscles.mp.

9) Neck muscles/

10) Neck extensor muscles.mp.

11) Cervical extensor muscles.mp.

12) Splenius muscles.mp.

13) 1 – 12 (OR)

14) Muscle Fatigue/

15) Fatigue/

16) Neuromuscular fatigue.mp.

17) Fatigability.mp.

18) Endurance.mp.

19) Exercise tolerance/

20) Task failure.mp.

21) 14 – 20 (OR)

22) Aging/

23) Healthy Aging/

24) Ageing.mp.

25) Aged/

26) Older people.mp.

27) Older adults.mp.

28) Elderly.mp.

29) Elders.mp.

30) 22 – 29 (OR)

31) **13 AND 21 AND 30 - TOT: 83 papers for MEDLINE – 253 papers for EMBASE**

**PUBMED**

(("Spine"[MeSH] OR "Spinal muscles" OR "Back Muscles"[MeSH] OR "Paraspinal Muscles"[MeSH] OR "Erector Spinae" OR "Multifidus" OR "Trunk Extensor Muscles" OR "Lumbar Extensor Muscles" OR "Neck Muscles"[MeSH] OR "Neck Extensor Muscles" OR "Cervical Extensor Muscles" OR "Splenius Muscles") AND ("Muscle Fatigue"[MeSH] OR "Fatigue"[MeSH] OR "Neuromuscular Fatigue" OR "Fatigability" OR "Endurance" OR "Exercise Tolerance"[MeSH] OR "Task Failure") AND ("Aging"[MeSH] OR "Healthy Aging"[MeSH] OR "Aged"[MeSH] OR "Older People" OR "Older Adults" OR "Elderly" OR "Elders" OR "Ageing")) - **TOT: 154 papers**

**Web of science**

<https://www.webofscience.com/wos/alldb/summary/edd1e4a9-175e-47c4-be0c-32deac02b1b9-0147ab5481/relevance/1>

(TS=("spine" OR "spinal muscles" OR "back muscles" OR "paraspinal muscles" OR "neck muscles"
OR "erector spinae" OR "multifidus" OR "trunk extensor muscles"
OR "lumbar extensor muscles" OR "neck extensor muscles"
OR "cervical extensor muscles" OR "splenius muscles"))
**AND**
(TS=("muscle fatigue" OR "fatigue" OR "neuromuscular fatigue" OR "fatigability"
OR "endurance" OR "exercise tolerance" OR "task failure"))
**AND**
(TS=("aging" OR "healthy aging" OR "aged" OR "older people"
OR "older adults" OR "elderly" OR "elders" OR "ageing")) and Preprint Citation Index (Exclude – Database) and Other or Review Article (Exclude – Document Types) and Dissertation Thesis or Awarded Grant or Data Set or Retracted Publication or Unspecified (Exclude – Document Types) and Patent (Exclude – Document Types) and Book (Exclude – Document Types)

**TOT: 734 papers**

**CINAHL Plus**

1) (MH "Spine")

2) "Spinal muscles"

3) "back muscles"

4) "Paraspinal Muscles"

5) (MH "Erector Spinae Muscles") OR (MH "Multifidus Muscles")

6) "Trunk Extensor Muscles"

7) "Lumbar Extensor Muscles"

8) (MH "Neck Muscles")

9) "Neck Extensor Muscles"

10) "Cervical Extensor Muscles"

11) "Splenius Muscles"

12) (MH "Muscle Fatigue")

13) (MH "Fatigue")

14) "neuromuscular fatigue"

15) "Fatigability"

16) "endurance"

17) (MH "Exercise Tolerance")

18) "task failure"

19) (MH "Aging") OR (MH "Healthy Aging")

20) (MH "Aged")

21) "Older People"

22) "Older Adults"

23) "Elderly"

24) "Elders"

25) "Ageing"

26) 1 OR 2 OR 3 OR 4 OR 5 OR 6 OR 7 OR 8 OR 9 OR 10 OR 11

27) 12 OR 13 OR 14 OR 15 OR 16 OR 17 OR 18

28) 19 OR 20 OR 21 OR 22 OR 23 OR 24 OR 25

29) 26 AND 27 AND 28

**TOT: 29 papers**

**TOT N PAPERS: 1253**
