# Supplementary material for: The effects of ageing on fatigue and endurance of the spinal extensor muscles: a systematic review and meta-analysis
Source: GeroScience. 2025 Nov 8;48(4):5785–809. doi: 10.1007/s11357-025-01987-x (PMC13575018; doi:10.1007/s11357-025-01987-x)
Supplement: Supplementary file 3 [file 11357_2025_1987_MOESM4_ESM.docx]

**Article title**: The effects of ageing on fatigue and endurance of the spinal extensor muscles: A systematic review and meta-analysis

**Journal name**: GeroScience

**Authors**: Martina Parrella^1^, Michail Arvanitidis^2^, Andrea Macaluso^1^, Deborah Falla^2^

**Affiliations**: ^1^Department of Movement, Human and Health Sciences, Università degli Studi di Roma “Foro Italico”, Rome, Italy.

^2^Centre of Precision Rehabilitation for Spinal Pain (CPR Spine), School of Sport, Exercise and Rehabilitation Sciences, College of Life and Environmental Sciences, University of Birmingham, Birmingham, UK.

**Corresponding author e-mail**: Professor Deborah Falla ([*d.falla@bham.ac.uk*](mailto:d.falla@bham.ac.uk))

**Supplemental file 4 Forest plot of endurance time differences between elderly and young adults including all 10 initially identified studies.** The plot displays mean differences (MD) in endurance time (seconds) with corresponding 95% confidence intervals (95% CI) for each included study. The green diamond represents the pooled effect estimate with its 95% CI, while the red line indicates the prediction interval. Negative values on the x-axis indicate decreased endurance time in elderly compared to young adults, while positive values indicate increased endurance in elderly. The size of each square is proportional to the study’s weight in the meta-analysis. The forest plot is organised in descending order based on publication year.
