# Supplementary material for: The effects of ageing on fatigue and endurance of the spinal extensor muscles: a systematic review and meta-analysis
Source: GeroScience. 2025 Nov 8;48(4):5785–809. doi: 10.1007/s11357-025-01987-x (PMC13575018; doi:10.1007/s11357-025-01987-x)
Supplement: Supplementary file 4 [file 11357_2025_1987_MOESM5_ESM.docx]

**Article title**: The effects of ageing on fatigue and endurance of the spinal extensor muscles: A systematic review and meta-analysis

**Journal name**: GeroScience

**Authors**: Martina Parrella^1^, Michail Arvanitidis^2^, Andrea Macaluso^1^, Deborah Falla^2^

**Affiliations**: ^1^Department of Movement, Human and Health Sciences, Università degli Studi di Roma “Foro Italico”, Rome, Italy.

^2^Centre of Precision Rehabilitation for Spinal Pain (CPR Spine), School of Sport, Exercise and Rehabilitation Sciences, College of Life and Environmental Sciences, University of Birmingham, Birmingham, UK.

**Corresponding author e-mail**: Professor Deborah Falla ([*d.falla@bham.ac.uk*](mailto:d.falla@bham.ac.uk))

**Supplemental file 5 Influence diagnostic plots for the meta-analysis of endurance time differences between elderly and young adults including all 10 initially identified studies.** The figure presents an influence analysis for the meta-analytic model, illustrating various diagnostic measures to identify influential studies. The plots include externally studentised residuals, DFFITS values, Cook's distances, covariance ratios, estimates of heterogeneity (τ² & Q-statistic) when each study is removed, hat values, weights and DFBETAS. The y-axis shows the values of each measure, and the x-axis displays the study numbers, ordered according to their appearance in the forest plot. The blue line in each plot represents the mean of each parameter across all studies.
